# Hashes are not suitable to verify fixity of the public archived web

**DOI:** 10.1371/journal.pone.0286879

**Published:** 2023-06-09

**Authors:** Mohamed Aturban, Martin Klein, Herbert Van de Sompel, Sawood Alam, Michael L. Nelson, Michele C. Weigle

**Affiliations:** 1 Computer and Mathematical Sciences Department, Columbia College, Columbia, Missouri, United States of America; 2 Los Alamos National Laboratory, Los Alamos, New Mexico, United States of America; 3 Data Archiving and Networked Services, The Hague, Netherlands; 4 Internet Archive, San Francisco, California, United States of America; 5 Department of Computer Science, Old Dominion University, Norfolk, Virginia, United States of America; Universiti Sains Malaysia, MALAYSIA

## Abstract

Web archives, such as the Internet Archive, preserve the web and allow access to prior states of web pages. We implicitly trust their versions of archived pages, but as their role moves from preserving curios of the past to facilitating present day adjudication, we are concerned with verifying the fixity of archived web pages, or mementos, to ensure they have always remained unaltered. A widely used technique in digital preservation to verify the fixity of an archived resource is to periodically compute a cryptographic hash value on a resource and then compare it with a previous hash value. If the hash values generated on the same resource are identical, then the fixity of the resource is verified. We tested this process by conducting a study on 16,627 mementos from 17 public web archives. We replayed and downloaded the mementos 39 times using a headless browser over a period of 442 days and generated a hash for each memento after each download, resulting in 39 hashes per memento. The hash is calculated by including not only the content of the base HTML of a memento but also all embedded resources, such as images and style sheets. We expected to always observe the same hash for a memento regardless of the number of downloads. However, our results indicate that 88.45% of mementos produce more than one unique hash value, and about 16% (or one in six) of those mementos always produce different hash values. We identify and quantify the types of changes that cause the same memento to produce different hashes. These results point to the need for defining an archive-aware hashing function, as conventional hashing functions are not suitable for replayed archived web pages.

## Introduction

Web archives, such as the Internet Archive (web.archive.org), UK Web Archive (webarchive.org.uk/ukwa/), and many others [1] have been established with the goal to preserve the web and allow access to prior states of web pages. One of the main reasons for archiving live web pages is that they often disappear or change over time due to the ephemeral nature of the web. If a web page disappears (e.g., returning “404 Not Found” from the live web) before it is captured by a web archive, then we will not be able to recover the missing web page or have any evidence of its existence in the past. An archived web page, or *memento*, is a resource on the web “that encapsulates a prior state” of a live web page [2], and is thus *fixed* at the date and time it was archived. However, we currently do not have any mechanism that allows us to verify the fixity of mementos. For example, if a web page was archived in 1999 and is replayed in 2022, how do we know that the archived version still encapsulates the state of the page as observed in 1999 and has not been modified or tampered with during those 23 years?

An example that highlights the importance of verifying the fixity of mementos is the story of Joy-Ann Reid, an American cable television host at MSNBC. In December 2017, she apologized for writing several “insensitive” LGBT blog posts nearly a decade earlier when she was a morning radio talk show host in Florida [3, 4]. In April 2018, Reid, supported by her lawyers, claimed that her blog and/or the archived versions of the blog in the Internet Archive had been compromised and the content was fabricated [5]. Even though the Internet Archive denied that their archived pages had been hacked [6], a stronger case could be made if we had an independent service verifying that those archived blog posts had not changed in the archive.

In the context of web archiving, *fixity* refers to the status of mementos being always fixed and unaltered. The TRAC report [7] indicates that verifying the fixity of archived content is needed to establish trust in digital repositories and archives. It is important for users of web archives, whether human or robot, to have the ability to verify the fixity of archived web pages for several reasons:

1. The number of public and private web archives is increasing [1, 8], and we may not have the same level of trust in all of these archives.
2. There is a current trend of using web archives for evidentiary purposes in court cases or to generally prove the existence of a web resource at a particular time in the past [4, 9–17].
3. There are different security threats against web archives [18–23] that not only affect accessibility to archived collections but also would change the representation of replayed archived pages over time. These concerns map to the Availability and Integrity components of the CIA security triad [24], respectively. Our main focus in this work is to aid in efforts to detect when Integrity is compromised.

A common technique for verifying fixity on digital resources is to generate a unique string, or a hash value, that represents the content of the resource at a particular time using cryptographic hashing algorithms, such as MD5 or SHA-256. The resulting hash values cannot be converted back to the original content. By periodically generating a new hash value on the content of a resource and comparing it with a previously computed and known to be correct hash, we can detect if the resource has been corrupted or altered.

**Fig 1:**
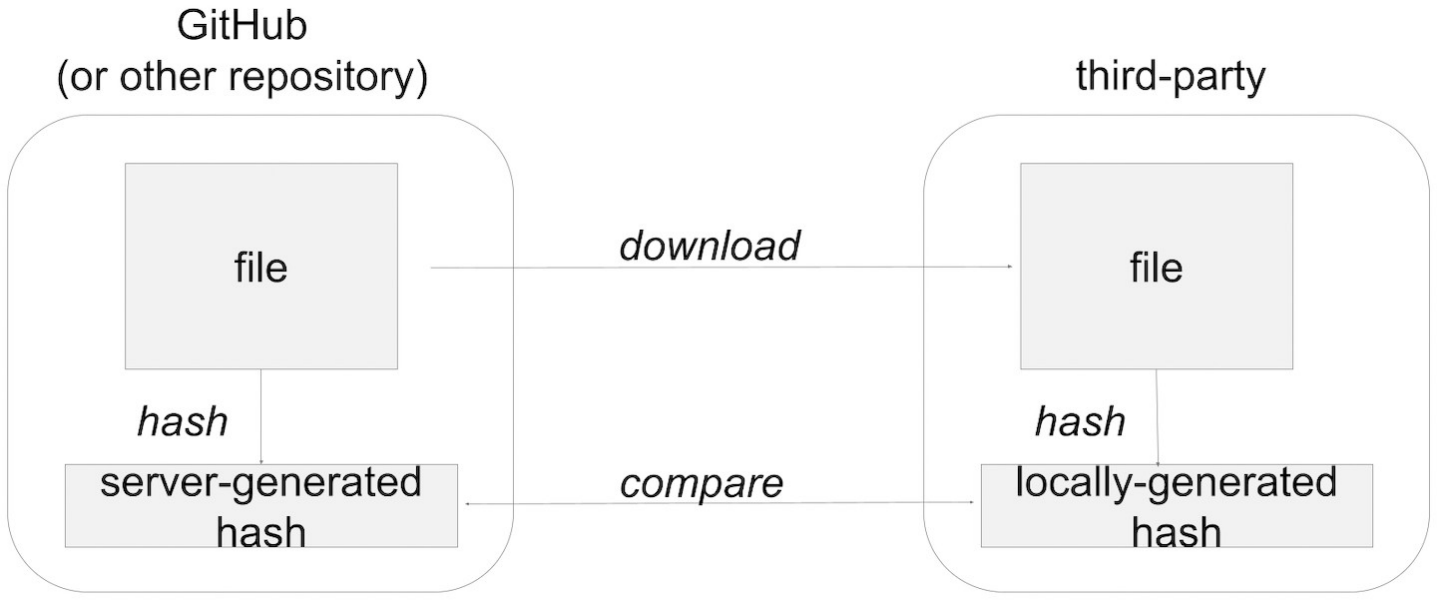
Verifying the fixity of a file in a GitHub repository. A third-party verifying the fixity of a file in a GitHub repository can directly download the file and access the associated server-generated hash to compare with a locally-generated hash.

Our goal in this work is to investigate the feasibility of computing fixity on *replayed mementos* rather than on the individual WARC files, which contain the original captured data and are located at a web archive. This concept differs from conventional approaches to utilize fixity information of digital files as frequently seen in the digital repository environment. For example, a repository such as GitHub that hosts a digital file also creates its content-based hash value and makes both available for download via the web (Fig 1). If a user is interested in verifying the fixity of the file, they can download it along with its corresponding hash value from GitHub. The user can then apply the same cryptographic hashing algorithm as GitHub to create a hash value of their own. If both hash values match, the user can be confident that the downloaded copy of the file is identical to the copy hosted by GitHub and has not been altered during the download process. This verification step is repeatable and, as long as the file is not altered, will always result in the same outcome.

**Fig 2:**
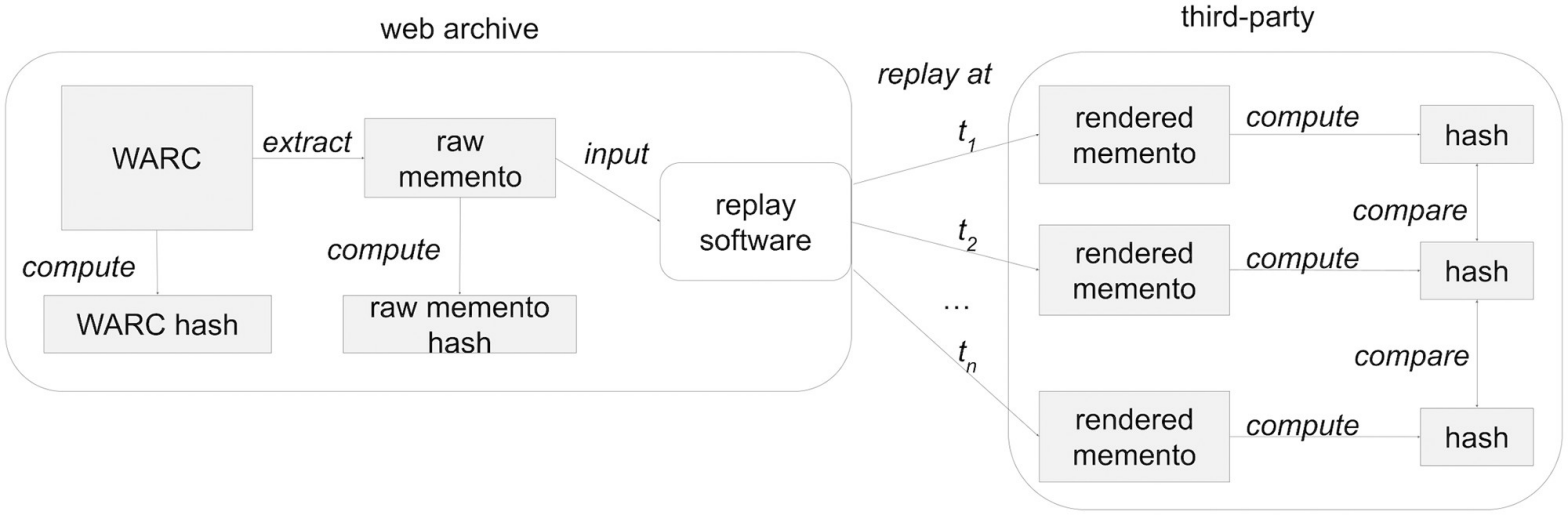
Verifying the fixity of a composite memento. A third-party verifying the fixity of an archived web page cannot directly download the WARC or the server-generated hash, but has to access the resource via replay software. We can compare multiple locally-generated hashes to each other, but there is no single server-generated hash available for comparison.

The picture is different for web archives since neither the raw WARC files nor their corresponding hash values are generally available to the public for download. In order to access the content of a memento, we therefore need to dereference its URI. This implies the involvement of archive-specific replay software that aims to present the archived web sources to the user at is was at the time it was captured. So rather than a simple file transfer via HTTP as in the GitHub scenario above, the replay software acts as an “in-between webserver” that extracts the memento from the WARC file and translates it into a resource that a web browser can display (Fig 2). In this scenario we are left with creating a hash value based on the content of memento as presented to us in a browser via the replay engine. In theory, the same immutable memento served via the same replay engine from the same web archive should result in the same content-based hash value, which would give us at least some sense of fixity. This process would allow users of web archives and independent third parties to verify that replayed archived resources have remained unchanged since their time of capture. Allowing verification by outside parties is important because if the contents of an archive are compromised, then the archive’s fixity information may also be compromised.

Some web archives make “raw” (i.e., unmodified) mementos available, which could be used to compute fixity. However, this is not a reliable proposition because: 1) not all archives support raw memento access, 2) since typical users do not interact with raw mementos, it would not represent a verification of a user’s experience, and 3) as we detail below, even raw mementos are sometimes transformed by web archives.

Our core finding presented in this paper is that generating repeatable hash values on replayed mementos is much more complicated, if not impossible, given the current landscape of replay software. We categorize and detail the reasons in the below:

- **Inclusion of Embedded Resources**: A memento generally consists of multiple resources embedded in the page. For example, the memento https://web.archive.org/web/20170717184643/https://climate.nasa.gov/vital-signs/carbon-dioxide/ consists of 201 images, 19 JavaScript files, 3 CSS files, and the base HTML file; if *any* of those embedded resources changes, one would say “the page has changed.” We refer to such a memento as a *composite memento*, representing the set of all resources comprising an archived page [25]. In this paper, we are interested in computing the fixity on a composite memento by generating one aggregated hash that represents the composite memento (cf. generating the hash value on only the base HTML file). This is challenging because any small change in the replay of the composite memento over time affecting either the base HTML file or any embedded resource will result in a different hash value.
- **Client-side Execution**: JavaScript, which runs on the client, can modify the Document Object Model (DOM) [26], dynamically adding or deleting HTML elements. JavaScript can also load more resources on a web page at any time after its initial loading. Even though two mementos may have the same JavaScript source code, the execution of the JavaScript may produce different results (e.g., based on random numbers or browser-specific information), and thus the pages may appear differently when rendered. Our prior work [27] found that about 54.5% of web pages in 2012 included JavaScript to load embedded resources, and the 2022 Web Almanac reports a median of 22 JavaScript files per desktop web page [28] and a median of 509 KB of JavaScript loaded for each desktop web page [29]. Thus, modern web pages contain a lot of JavaScript, and executing JavaScript code impacts generating repeatable hash values on composite mementos.
- **Link Modification**: Web archives transform captured web pages to appropriately replay them in a user’s browser. (Berlin [30, 31] describes the various ways in which archives modify mementos.) Part of the transformation process includes rewriting links of embedded resources so that these resources are retrieved from the archive, not from the original server. For example, the logo image https://media.npr.org/chrome_svg/npr-logo-color.svg that is embedded in the memento https://web.archive.org/web/20180618094908/https://www.npr.org/ is rewritten as https://web.archive.org/web/20180618094908im_/https://media.npr.org/chrome_svg/npr-logo-color.svg, but when the browser dereferences that SVG URL, the web archive will redirect the browser to the memento that is “closest” in time, in this case, https://web.archive.org/web/20180618095215im_/https://media.npr.org/chrome_svg/npr-logo-color.svg. Typically, resources within the same composite memento are not captured by the archive at the same time. For example, from the links above, there is a difference (about 3 minutes) between the time when the base HTML file is captured by the archive (Memento-Datetime: June 18, 2018 09:49:08 GMT) and when the embedded logo image is captured (Memento-Datetime: June 18, 2018 09:52:15 GMT). At replay time, all URLs are rewritten relative to the base HTML (in the above example, 20180618094908) and when dereferenced, the archive redirects to the temporally closest available memento. This delta in Memento-Datetime can range from zero seconds to many years, and when resources are shared between multiple pages, such as style sheets and images (both of which typically change more slowly than text resources), large deltas are common as a result of the archives optimizing their crawl strategies. (Large Memento-Datetime deltas themselves are not the problem, for example a GIF that never changed after it was published would remain valid even if the delta was measured in years, but a dynamically updated GIF could be invalid even if the delta is measured in seconds [25, 32].) The holdings of a web archive are dynamic, and new resources, with varying values of Memento-Datetime, are accessioned all the time, and some resources are even occasionally lost, which means that the deltas can vary over time. In summary, the web archive policy of rewriting embedded resources relative to the Memento-Datetime of the base HTML file and then doing a best effort resolution at the time of replay can result in changes in the composite memento even when the base HTML has not changed.
- **Inclusion of Archive-specific Resources**: Archives may add their own archival banners in the base HTML of a memento to provide metadata about both the memento being viewed and the original page. Including such archive-specific content (e.g., archival banners), which often contains dynamic information reflecting the current state of the archive, in the hash calculation affects generating repeatable hashes.

In this paper, which is based on Aturban’s PhD thesis [33], we wanted to test the standard digital preservation technique of using hashes for verifying fixity. We selected 16,627 mementos from 17 public web archives. Over a period of 14 months, we downloaded each memento 39 times and computed their hash values on each replay (for a total of 648,453 hash calculations). For the reasons outlined above, we expected there would be challenges and that not all mementos would produce stable hash values through time. However, we were surprised at the extent of the changes we discovered: the fixity of the replayed pages changed often enough to effectively negate the utility of conventional hashing techniques for third-party auditing of archived web pages.

Through the downloaded content of the mementos and their resulting hash values, we provide answers to the following questions:

- What are the types of changes in the playback of composite mementos that prevent or affect generating repeatable hashes? We compare the resulting 39 consecutive hash values for each composite memento. Each time two consecutive hashes are not identical, we look at the downloaded content to find out which resource (or resources) is causing different hash values.
- How many times does each identified type of change occur? This will help us in the future in defining requirements for generating repeatable hash values.
- For each composite memento, will excluding certain embedded resources that we expect to change over time help to generate repeatable hash values? Would, for example, excluding resources added by the archive produce more consistent hash values?

## Background and related work

In this section we briefly explain how live web pages are rendered, and how archives crawl the web and replay mementos. Then, we review related work in trusted timestamping of digital resources, including trusty URIs. Next, we define fixity in the context of digital preservation and why it is important to verify fixity of mementos. Finally, we describe several security issues in web archives with relevance to our work. Although we cover related work in verifying fixity on digital resources, we emphasize that there are no existing solutions to computing or verifying fixity on composite mementos.

### Rendering live web pages

HyperText Markup Language (HTML) is the standard markup language that specifies how web pages are structured using a set of defined tags and tag attributes. Web pages written in HTML are accessed from servers via the HTTP protocol. A web browser sends an HTTP request to the server. HTTP request headers are used to support HTTP content negotiation so that the client (e.g., the browser) can indicate which content is preferred, such as a document in a specific language or format. The server handles the HTTP request and sends back an HTTP response to the browser. The returned content includes HTTP response headers and the requested HTML file.

Upon receiving the response, the browser renders the returned HTML. In order for the browser to display the entire web page, it will request other resources, such as images, style sheets, and JavaScript files, specified in the HTML by tags like <img>, <link>, and <script>. The number of resources embedded in a web page varies from zero to even hundreds. For example, the front page of CNN (https://www.cnn.com) as of March 2023 contained almost 200 embedded resources. The 2022 Web Almanac reports that the median number of requests to construct a desktop web page is 76 [29]. Web resources comprising a web page might be served from the same server hosting the base HTML file or from any other web server. In general, in response to an HTTP request, a server returns an HTTP response that should consist of the following:

1. The HTTP status code: The status code indicates whether the HTTP request has been successfully handled by the server. For example, the status code 404 Not Found indicates that the requested resource could not be found in the server while 200 OK indicates that the server completed the request.
2. The HTTP response entity headers: The headers contain information about the payload (e.g., Content-Length and Content-Type), the client/server connection (e.g., Keep-Alive and Connection), or the server (e.g., server).
3. The HTTP response entity body: The body is the response payload (i.e., the content of the requested resource). An HTTP response may not contain an entity body, for instance for responses with the HTTP status code 304 Not Modified.

### Web archiving

Web archiving is the process of preserving portions of the current web for future generations. Web archiving is not only concerned with collecting and preserving web pages but also with how to provide access to those archived resources. Web archives hold billions of archived web pages [34]. For instance, we expect to find archived copies of a well-known web page (e.g., www.cnn.com) in large web archives, such as the Internet Archive, as these archives try to capture the entire web by employing large-scale web crawlers. Other web archives focus on preserving special collections. For instance, the UK Web Archive was established with the objective of archiving only UK websites (e.g., www.parliament.uk) [35]. Other web archives, such as perma.cc, webcitation.org, and archive.is, capture web pages on demand, so they only preserve pages submitted by users, not through crawling the web. Gomes et al. [36] developed a survey of various web archiving initiatives as of 2011, and the authors created a Wikipedia page [37] that has been used to keep the information updated. Table 1 shows a list of the 17 public web archives used in our study.

**Table 1:** Our set of 17 public web archives.

Archive URI	Archive Name
swap.stanford.edu	Stanford Web Archive Portal
web.archive.org	The Internet Archive
archive.bibalex.org	Bibliotheca Alexandrina’s Internet Archive
arquivo.pt	The Portuguese Web Archive
collectionscanada.gc.ca	Library and Archives Canada
digar.ee	The Estonian Web Archive
nationalarchives.gov.uk	The UK National Archives
vefsafn.is	The Icelandic Web Archive
webarchive.loc.gov	Library of Congress Web Archives
webarchive.org.uk	The UK Web Archive (UKWA)
webarchive.proni.gov.uk	Public Record Office of Northern Ireland (PRONI)
webharvest.gov	US Congressional & Federal Government Web Harvests
archive-it.org	Archive-It—Web Archiving Services for Libraries and Archives
archive.is	Archive.is
perma.cc	Perma.cc
webcitation.org	WebCite
europarchive.org	The European Archive

### Memento protocol

Memento [2, 38] is an HTTP protocol extension that uses time as a dimension to access the web by relating current web resources to their prior states. The Memento protocol is supported by most public web archives including the Internet Archive. The protocol introduces two HTTP headers for content negotiation. First, Accept-Datetime is an HTTP Request header through which a client can request a prior state of a web resource by providing the preferred datetime (e.g., Accept-Datetime: Mon, 09 Jan 2017 11:21:57 GMT). Second, the Memento-Datetime HTTP Response header is sent by a server to indicate the datetime at which the resource was captured. The Memento protocol also defines the following terms:

- URI-R—an original resource from the live Web
- URI-M—an archived version (memento) of the original resource at a particular point in time
- URI-T—a resource (TimeMap) that provides a list of mementos (URI-Ms) for a particular original resource

A Memento aggregator can be used to retrieve TimeMaps aggregated from multiple web archives. The Memento Aggregator from Los Alamos National Laboratory (LANL) [39, 40] is one implementation that provides TimeMaps aggregated from different web archives both with (a) native support of the Memento protocol and (b) by proxy support of the Memento protocol. MemGator [41, 42] is an open-source implementation that provides a variety of customization options, such as allowing users to specify a list of web archives to retrieve TimeMaps from, but it only aggregates TimeMaps from archives that natively support the Memento protocol.

### Crawling and replaying archived web pages

A web crawler is an automated program that is used by web search engines (e.g., Google and Bing) and web archives to systematically collect and discover web pages (URIs) that exist on the web. The main purpose of crawling the web by search engines (e.g., via Google Googlebot [43]) is to index web pages and understand what the content of each page is about to be able to respond to users with the most relevant web pages to their queries. Web archives use web crawlers, such as the Internet Archive’s Heritrix [44], to collect and preserve web pages, and allow access to those archived pages. In general, an archive’s web crawler performs the following (simplified) steps to crawl live web pages:

1. Insert a given set of URIs (i.e., seed URIs) in a queue.
2. Select (or dequeue) one URI from the queue.
3. Dereference the web page identified by the selected URI.
4. Write the downloaded content of the web page to a file. The most common file format used by web archives is Web ARChive (WARC) [45], which specifies a set of rules for aggregating multiple web resources (e.g., HTML files, images, and style sheets) with the HTTP request/response entity and headers of each resource in addition to WARC-related metadata into a single file. WACZ [46], a recent extension of WARC that aggregates indexes with the associated WARC contents, is similar in purpose.
5. Extract any new URIs that have not yet been crawled or placed in the queue from the downloaded content of the page.
6. Insert the newly discovered URIs in the queue.
7. If the queue is not empty, go to step 2.

The crawling process will result in a set of archived pages. To provide access to their archived pages, many web archives use OpenWayback [47], the open-source implementation of IA’s Wayback Machine, to allow users to look up archived pages by submitting URIs. On the replay of an archived page, one of the main tasks of OpenWayback is to ensure that all resources comprising the page (e.g., images, style sheets, and JavaScript files) are retrieved from the archive, not from the live web. Thus, at the time of replaying the page, OpenWayback rewrites all links to those embedded resources to point directly to the archive [48]. In addition to OpenWayback, PyWB [49] is another replay tool, which is used by Perma [50], Webrecorder [51], and an increasing number of web archives, as the International Internet Preservation Consortium (IIPC) recommended in 2020 [52] that its members to transition to PyWB. Although the Internet Archive’s Wayback Machine is proprietary software, both OpenWayback and PyWB mimic its design and functionality. Despite the multiple software implementations, the Wayback Machine model of archival replay, using WARC files as input, is the predominant model in public web archives; of the 17 web archives listed in Table 1, only archive.is does not use WARC files and Wayback Machine modalities. While the Wayback Machine model of replay has clearly been successful, researchers have begun to consider how the standardization of WARC files and the Wayback Machine model itself have shaped the field of web archiving (e.g., [53, 54]). One of the key assumptions in the Wayback Machine model is that HTTP responses are stored in WARC files, and then replayed through the web archive with a mixture of client-side and server-side transformations to both recreate the past (e.g., rewrite links to point back into the web archive) and “brand” the replayed content as the past web (e.g., archival banners). Although some web archiving projects have been introduced that break with this model of Wayback Machine archival replay—such as Jawa [55], which outright eliminates JavaScript that can be detrimental to replay, and OldWeb.today [56], which emulates a browser within the actual user’s web browser and then replays the web content with no transformations at all—the Wayback Machine model of partial transformation remains what users are most likely to encounter when they go to “a web archive.”

To illustrate how web archives transform the content of an original web page to appropriately replay it in a user’s browser, we submitted the web page’s URI https://maturban.github.io/playground/index.html to the Internet Archive using the “Save Page Now” feature [57]). The archive captures the root HTML file and all embedded resources included in the page. Table 2 shows the URI-Rs of the original resources comprising the web page and the corresponding URI-Ms to the mementos created by the Internet Archive (the archival datetime of each memento is marked in red).

**Table 2:** URI-Rs and URI-Ms of example web page. The URI-Rs of the original resources and the URI-Ms of their corresponding mementos for the web page https://maturban.github.io/playground/index.html.

Original page (URI-R)	Memento (URI-M)
https://maturban.github.io/playground/index.html	https://web.archive.org/web/20190725212938/https://maturban.github.io/playground/index.html
https://maturban.github.io/playground/styles.css	https://web.archive.org/web/20190725212938/https://maturban.github.io/playground/styles.css
https://www.odu.edu/images/logo-university.png	https://web.archive.org/web/20190725212938/https://www.odu.edu/images/logo-university.png

**Fig 3:**
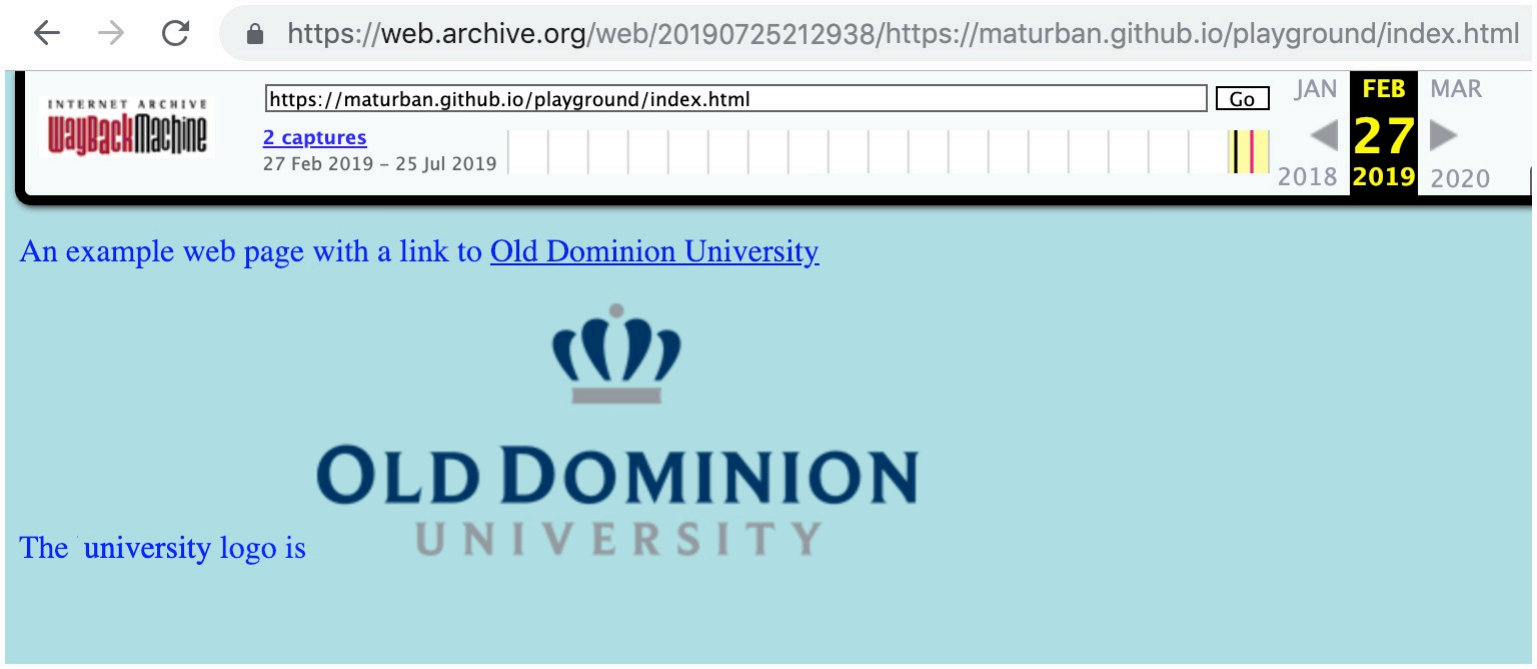
The representation of the memento https://web.archive.org/web/20190725212938/https://maturban.github.io/playground/index.html. M. Aturban, A Framework for Verifying the Fixity of Archived Web Resources, PhD dissertation, 2020.

Fig 3 illustrates the representation of the memento replayed in a web browser. The archive transformation process of the original page may include injecting additional HTML elements and rewriting all links of embedded resources so they point to the archive, not to the live web. Archives also add banners [58] to provide information about both the memento being viewed and the original page (e.g., the top portion in Fig 3).

**Fig 4:**
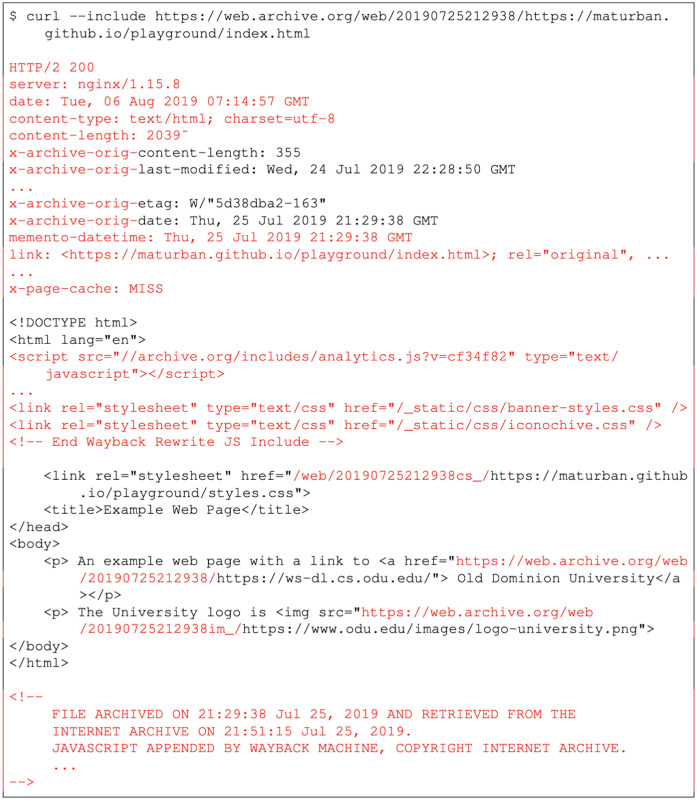
The rewritten HTML of the memento https://web.archive.org/web/20190725212938/https://maturban.github.io/playground/index.html. The code marked in red was added by the archive. The archive also modifies the names of original headers by adding x-archive-orig at the beginning of these headers. M. Aturban, A Framework for Verifying the Fixity of Archived Web Resources, PhD dissertation, 2020.

As illustrated in Fig 4, we used cURL to download the memento. The archive-specific code is marked in red. Archives may prepend the string X-Archive-orig- to the original HTTP response headers (i.e., the headers returned by the server from which the original page is captured). Therefore, users can differentiate between the original response headers and the response headers that are added by the archive (e.g., Memento-Datetime). In Fig 4 we also show the HTML that is returned by the web archive. The items in red were added/modified by the archive. Note the rewritten URIs, as listed in Table 2.

Archives may create multiple mementos for the same original resource at different times. During replay of a memento, a number of embedded mementos might not be available in the archive due to technical or performance issues. In this case, the archive will try to use the closest available memento instead. For example, if for some reason the 20190725212938 (Memento-Datetime: Thu, 25 Jul 2019 21:29:38 GMT) university logo (Table 2) is unavailable, the Internet Archive Wayback Machine will not return an HTTP 404 Not Found, but instead will issue an HTTP 302 Found redirect to the 20190725054927 (Memento-Datetime: Thu, 25 Jul 2019 05:49:27 GMT) version of the university logo because it is the temporally closest copy. (The full TimeMap for this URI-R can be found at: https://web.archive.org/web/timemap/link/https://www.odu.edu/images/logo-university.png.) The exact URI-M of embedded resources can silently vary on each replay of a composite memento, since the design of the Wayback Machine is to always redirect to the temporally closest memento, if one exists, if it does not have exact requested Memento-Datetime.

**Fig 5:**
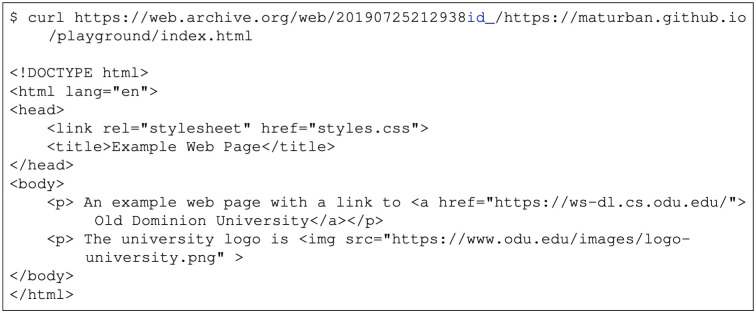
The raw HTML from requesting the memento https://web.archive.org/web/20190725212938id_/https://maturban.github.io/playground/index.html. M. Aturban, A Framework for Verifying the Fixity of Archived Web Resources, PhDdissertation, 2020.

In addition to the rewritten content, many archives allow accessing unaltered, or raw, archived content (i.e., the original content without any type of transformation by the archive). Jones et al. [59–61] explore transformation of original content performed by different web archives and introduce several rules for acquiring raw mementos. The most common mechanism to retrieve a raw memento is by adding id_ [62, 63] after the timestamp in the requested URI-M as Fig 5 shows. Since a raw memento has no link modification, if it is loaded in a web browser, all of the embedded resources will be requested from the live web. In many cases, these resources no longer exist, so the page will not render as expected. The intention and expectation of raw access is that the replayed resources do not change from how they were first archived. In practice, we saw this was not always the case; Section “Quantifying the Types of Changes” details several scenarios where web archives modified the responses even when raw mementos were requested.

### Trusted timestamping of digital resources

Timestamping is recording the date and time of when an event occurs. A “trusted” timestamp is a timestamp initially created and verified by a trustworthy third-party service. Several tools have been developed to generate trusted timestamps. For example, OriginStamp [64] allows users to generate a trusted timestamp using blockchain-based networks on any file, plain text, or a hash value. The data is hashed in the user’s browser and the resulting hash is sent to OriginStamp’s server to be added to a list of all hashes submitted by other users. Once per day, OriginStamp generates a single aggregated hash of all received hashes. This aggregated hash is converted to a Bitcoin address that will be a part of a new Bitcoin transaction. The timestamp associated with the transaction is considered a trusted timestamp. A user can verify a timestamp through OriginStamp’s API or by visiting their website.

Other services, such as Chainpoint (chainpoint.org) and OpenTimestamps (opentimestamps.org), are based on the same concept of using blockchain-based networks to timestamp digital documents. Even though users of these services can pass data by value, they are not allowed to submit data by reference (i.e., passing a URI of a web page). In other words, these tools *cannot* be used to timestamp web pages. The only exception is a service [65] established by OriginStamp that accepts URIs from users, but the service is no longer available on its prior live web address of (https://www.isg.uni-konstanz.de/web-time-stamps/, so we cannot evaluate how well it worked.

### Verifying the fixity of digital resources

In the context of digital preservation, fixity is a mechanism to demonstrate that archived resources have remained unaltered since the time they were captured [66]. The final report of the PREMIS Working Group [67] defines information used for fixity as “information used to verify whether an object has been altered in an undocumented or unauthorized way.”

To establish trust in repositories and web archives, different publications and standards have emphasized the importance of verifying fixity of archived resources. The Trusted Repositories Audit & Certification (TRAC) report [7] by the Task Force on Archiving of Digital Information introduces criteria for identifying trusted digital repositories. In addition to the ability to reliably provide access, preserve, and migrate digital resources, digital repositories, which include web archives, must create preservation metadata that can be used to verify that content is not tampered with or corrupted (fixity) according to Sections B2.9 and B4.4. The report recommends that preserved content is stored separately from fixity information, so it is less likely that someone is able to alter both the content and its associated fixity information [7].

As illustrated earlier in Fig 2, many web archives compute and store hashes of their archival content. The Internet Archive creates and makes available both the hash of an entire WARC file and the hash of raw mementos (more accurately, the WARC Payload Digest [45]). WARC file hashes are available via the Internet Archive’s Web Crawls interface, https://archive.org/details/web, which provides access to metadata about various web crawls stored at the Internet Archive. This metadata includes MD5 and SHA1 hashes on the individual WARC files generated by a crawl.

The hashes of raw mementos are stored and made available via the Internet Archive’s CDX API [68]. However, this hashing of the WARC Payload Digest is sensitive to content-encoding [69]. That is, if the payload stored was received as compressed (e.g., GZip or Brotli [70]), then the payload digest would be different than if it were in plain text, even if the content served to the client at replay time would be the same (as any stored content-encoding is undone at replay time). This is because different archiving tools (e.g., Heritrix, Wget, Zeno [71], and Brozzler [72]) each have their own preferences of content negotiation, which affects the content-encoding that is stored. The result is that two mementos with the same content may produce different raw memento hashes based on the tool they were archived with. Further, even the most basic change to the raw memento by replay (via URL rewriting) will lead to a different hash being computed on the client-side. This implies that comparing the original raw memento hash and the replayed memento hash would not be effective (for some media types) and other approaches should be explored.

Generating *repeatable* fixity information and using it to ensure that archived resources are valid will help to establish trust in web archives. Part of the problem, though, is the lack of standard techniques that users can apply to verify the fixity of replayed archived web pages [73–75]. Jinfang Niu [76] mentioned that none of the web archives declare the reliability of the archived content they preserve, and some archives, such as the Internet Archive and Government of Canada Web Archive, have a disclaimer [77] stating that they are not responsible for the reliability of the archived resources.

Kuhn et al. [78] define a trusty URI as a URI that contains a cryptographic hash of the content it identifies, thereby associating the name (URI) of the page with its content (hash). With the assumption that a trusty URI, once created, is linked from other resources or stored by a third party, it becomes possible to detect if the content that the trusty URI identifies has been tampered with or manipulated on the way (e.g., to prevent man-in-the-middle attacks [79]). In their second paper [80], Kuhn et al. introduce two different modules to allow creating trusty URIs on different kind of content, but this is limited to RDF graphs and byte-level content. No modules have been introduced for HTML documents or for complete web pages (i.e., computing hashes on a base HTML file and all its embedded resources).

In theory, trusty URIs should work for raw (unrewritten and unexecuted) composite mementos. As described in Kuhn et al. [78], trusty URIs could be computed for each embedded resource, and then a trusty URI would be computed for the enclosing resource (that contained references to the embedded resources). However, there are several reasons why this is an unlikely solution for web archives. First, the various web archives already have their established URI-M naming conventions, and placing the hash value of the HTTP entity in the URI-M itself would represent a significant engineering and branding change that currently seems unlikely to be made. Second, computing fixity on HTML pages would be difficult: replayed HTML changes over time, both by including the date and other replay metadata in the HTML comments (effectively making each replay unique), as well as by evolving JavaScript injected into the HTML for improved replay (e.g., JavaScript improvements that allowed cnn.com to be successfully replayed after approximately four years of failure [81]). Third, if fixity was computed on raw resources, the links to those resources would have to be rewritten, meaning they would no longer be raw. Fourth, as will be shown in Section “Archive-Level Changes”, embedded resources often change on each replay, so computing fixity on a composite memento is not straightforward (i.e., even if the fixity of individual embedded resources can be verified, the set of embedded resources comprising the composite memento will change). Lastly, we would like to be be able to compute fixity on archived pages without depending on their opting into trusty URIs.

There have been several exploratory studies regarding publicly storing the hash values of archived resources in either blockchains or in other web archives. All such projects remain proofs-of-concept and have not seen widespread adoption. In the ARCHANGEL project [82, 83], Collomosse et al. compute hashes on digital documents and store them in the Ethereum blockchain. The documents are not necessarily stored in web archives, and the emphasis appears to be on documents that can be expected to remain static (e.g., video files). In the WARChain project [84, 85], hashes are computed server-side by the web archive and then stored in the EduPoS blockchain. The hashes are computed on the text in HTML documents, as extracted by the popular BeautifulSoup library [86]. Neither ARCHANGEL nor WARChain attempt to deal with the complexities of composite mementos. In the Synchronic Web project [87], simultaneous publishing web content and hashes of the web content in a blockchain is proposed. The use of this blockchain content has been proposed to support web archives [88], but while performance has been evaluated, to date no consideration has been made in how the playback interface transforms the content (i.e., it is suitable for the scenario depicted in Fig 1 but not the web archive scenario depicted in Fig 2). In our prior work on the Archival Fixity Server project [89], we utilize an *archival fixity server* that publishes manifests for individual URI-Ms and how their hashes were computed (so they can vary per resource). These manifests are then given trusty URIs (so the manifest’s integrity can be verified), and then the manifests are pushed into multiple, different web archives. This allows for verification of individual URI-Ms, but does not address how a set of URI-Ms were assembled into a composite memento for a particular replay of an archived page. In other words, even if we can verify the integrity of the individual resources, there is no mechanism to verify that a particular set of archived resources in a composite memento was ever witnessed in the past (cf. temporal violations [32]).

### Web archiving security issues

Few scholars have focused on security issues related to web archives and replay of mementos. To emphasize the importance of verifying the fixity of archived pages, we briefly summarize some related efforts below. For a more detailed overview and discussion, we refer to Aturban’s PhD thesis [33]. Lerner et al. [90] discovered several vulnerabilities in the Internet Archive’s Wayback Machine that could be leveraged to modify the rendered memento in a browser and therefore prevent generating repeatable fixity information on replayed mementos:

- **Archive-Escapes**: Some web links are generated by JavaScript at replay time and therefore not rewritten by the archive, which leads to them being loaded from the live web. Whoever owns and controls the original server that hosts the live resource linked from the archive can inject malicious code (e.g., JavaScript) to change the client’s rendered view of the archived page. Brunelle et al. [91] provided some additional examples that illustrate the effect of live resources linked from archived pages.
- **Same-Origin Escapes**: Malicious code in a third-party <iframe> could be ingested into a web page before it is archived. While this <iframe> cannot modify the main HTML file on the live web due to the Same-Origin Policy, the Policy becomes ineffective once the page is archived and the main HTML file as well as the <iframe> are loaded from the archive’s domain for replay. Both scenarios can also be combined for malicious purposes.
- **Anachronism-Injection**: If a composite memento contains a resource that has never been captured by the archive, whoever has access to the original server hosting that never-archived resource can publish a malicious version of it on the live web and submit it to the archive. The archive will capture the malicious resource and embed it in the composite memento.

The Internet Archive, in response to Lerner’s suggestions, addressed some of these issues by establishing the Content-Security-Policy HTTP header [18] that notifies a user-agent (e.g., a web browser) to not load resources except from specified domains (e.g., an archive’s domain). However, the problem of having live web resources linked from archived pages may still occur in the Internet Archive and other archives because there are several methods that can load live web resources into archived pages as explained by Nelson [19, 23].

Cushman and Kreymer [92, 93] created a shared repository [94] of seven potential threats in web archives. Some of these threats, for example, the Archive-Escapes scenario, overlap with those highlighted by Lerner et al. [90]. However, additional threats outlined by Cushman and Kreymer that may affect the ability to generate repeatable fixity information for mementos are:

1. **Showing different page contents when archived**: An original server can notice when a web page is being crawled by an archive. At this time, the server can reply with content that is different from the content of the page it would otherwise serve. It is also technically possible that a page is designed so that it knows when it is being replayed from an archive, and shows different content, accordingly.
2. **Banner spoofing**: Malicious code can be used to change the appearance of the archive’s banner. This scenario is similar to Lerner et al.’s Archive-Escapes but focuses on web archives’ specific banner.

Rosenthal et al. [95] described several threats against the content of digital preservation systems such as web archives. The authors argue that designers of archives must be aware of threats, such as media failure, hardware and software failure, communication errors, failure of network services, etc.

Watanabe et al. [96] described many of the same vulnerabilities and possible remedies as Lerner et al. [90] and Cushman and Kreymer [94], but provided a broader study of “web rehosting” services. Out of the 21 total rehosting services they investigated, 18 had at least one vulnerability. These rehosting services include privacy proxies (e.g., proxysite.com), language translation services (e.g., translate.google.com), and web archives, all of which have the basic structure of taking the URL of a page and passing it as an argument to a service at another URL.

## Methods

The main goal of our work is to observe the playback of mementos over time and try to understand the challenges in generating repeatable fixity information (e.g., hash values) on replayed mementos. Our initial assumption was that mementos should not change over time, or in other words, we should always be able to calculate the same hash each time the same memento is downloaded. However, we found that this is not always the case. To study this further, we wanted to identify and quantify the types of changes that we observed.

**Fig 6:**
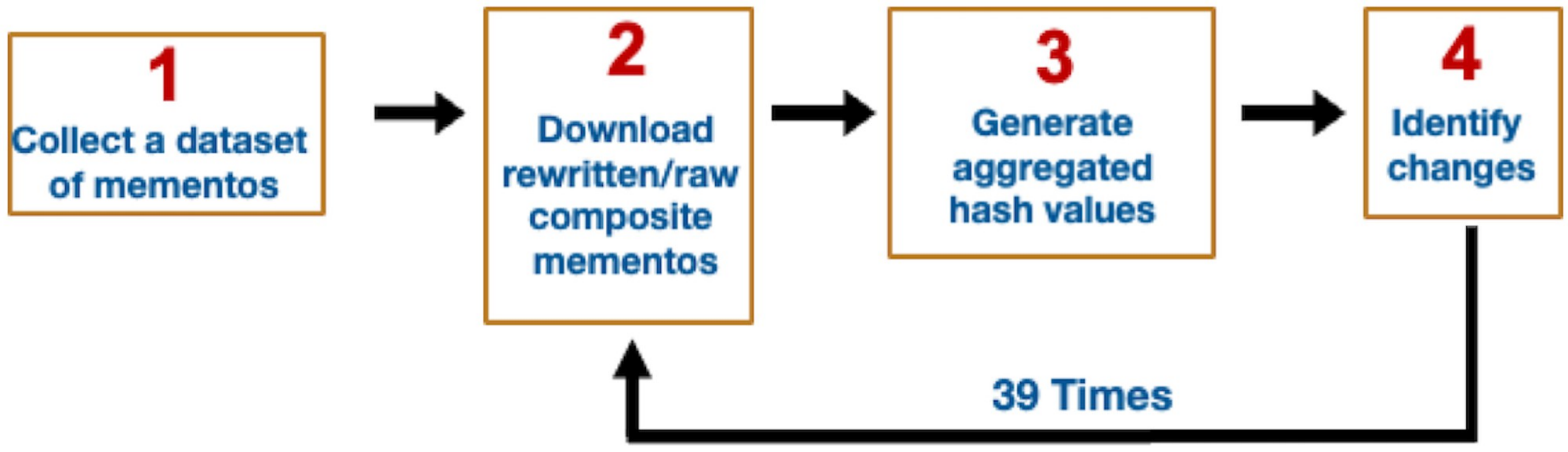
Overview of our methodology for evaluating the use of hashes for determining fixity on replayed mementos.

We selected mementos from the 17 public web archives listed in Table 1 and downloaded each memento on 39 different days. To determine if the memento had changed over time, we used Merkle trees [97] to generate a single hash value for each downloaded memento and used these hashes to identify when there were differences for the same memento. Fig 6 shows an overview of our general process. We will briefly discuss each of the steps 1–3 here (step 4 will be discussed in the next section). Additional details on our methodology and results are available in Aturban’s PhD thesis [33].

Each memento we evaluate is actually a composite memento, made up of the base HTML page and all embedded resources used to render the page. We compute hash values on all of the elements of the composite memento, including a selection of the HTTP response headers (those that should not change) and the URI-Ms of the resources themselves. To set a baseline, we compute hashes on the content of raw memento resources (without archive modification). To account for additional resources loaded at playback time (e.g., those loaded by JavaScript), we also compute hashes on the replayed content. All of these hashes are combined into a single hash for the composite memento using Merkle trees, as will be described later. This hash can be viewed as a value that summarizes the memento’s content at a particular point in time. When there are differences in consecutive hashes, the use of Merkle trees will allow us to analyze the individual components to help determine where the change occurred.

### Step 1: Collect a dataset of mementos

In November 2017, we collected a dataset of 16,627 mementos of 3,698 unique URI-Rs (original resources) from the 17 public web archives shown in Table 1. Our technical report [98] describes in detail the methods we used to create this dataset. We provide a summary of the process here.

We wanted to build a dataset of mementos that spanned a wide range of web page types and web archives, including those that used different playback software. We set some initial collection targets for URI-Rs:

- at least 200 URI-Rs per archive
- include some well-known web pages
- include web pages at multiple path lengths (i.e., some top-level, some deep links)
- include web pages likely to contain embedded resources, such as images, CSS, JavaScript

**Fig 7:**
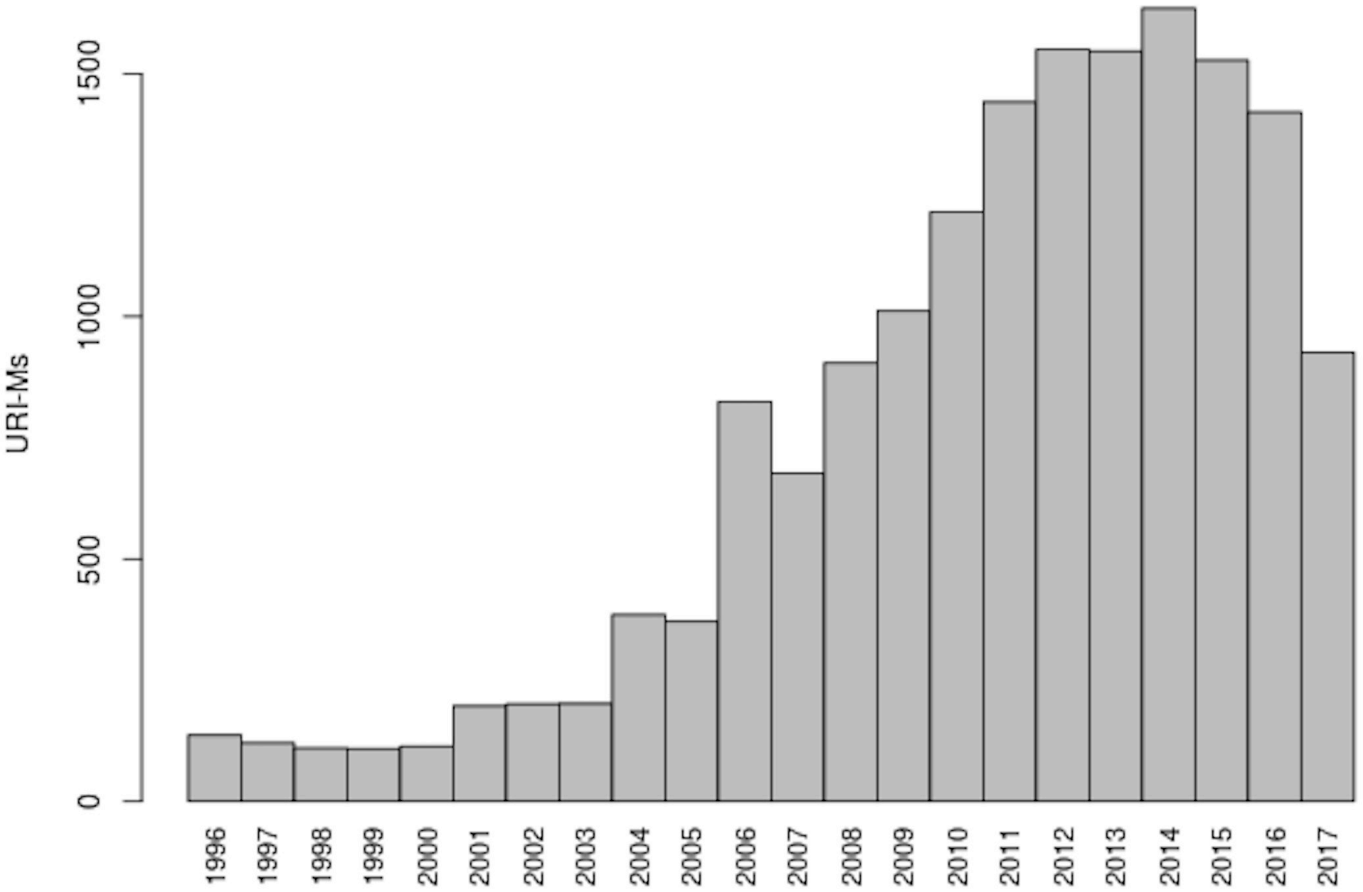
URI-Ms collected per year. Note that we collected mementos on November 15, 2017, and thus, the number of mementos from 2017 is fewer than the number of mementos in other years. M. Aturban, A Framework for Verifying the Fixity of Archived Web Resources, PhD dissertation, 2020.

Table 3 shows the final numbers of selected URI-Rs and URI-Ms per archive, and Fig 7 shows the number of URI-Ms collected per year.

**Table 3:** Final URI-Rs and URI-Ms selected per archive. The 16,267 total URI-Ms come from 3,698 unique URI-Rs. We include some of the same URI-Rs in multiple archives because they produce different URI-Ms.

Archive	URI-Rs	URI-Ms
web.archive.org	1,566	1,566
archive-it.org	1,338	1,383
archive.is	1,257	1,396
webarchive.loc.gov	1,059	1,594
arquivo.pt	766	1,569
webcitation.org	720	1,585
europarchive.org	321	979
swap.stanford.edu	302	1,222
vefsafn.is	290	1,589
webharvest.gov	247	712
digar.ee	225	488
webarchive.org.uk	221	349
webarchive.proni.gov.uk	209	469
nationalarchives.gov.uk	200	994
collectionscanada.gc.ca	198	351
perma.cc	175	182
archive.bibalex.org	168	199

**Fig 8:**
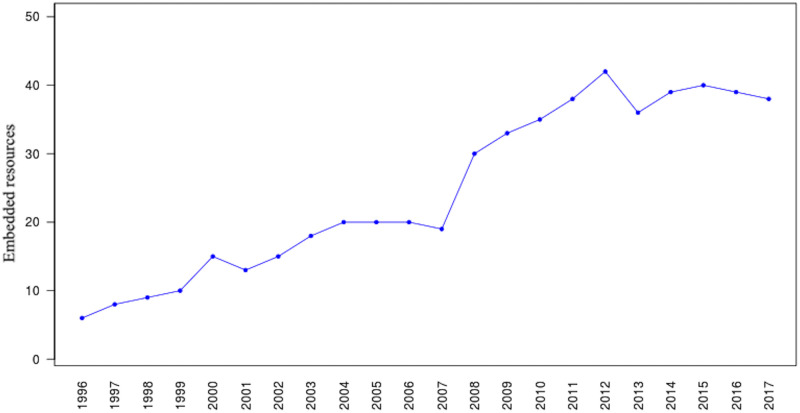
Median number of embedded resources per memento per year. M. Aturban, A Framework for Verifying the Fixity of Archived Web Resources, PhD dissertation, 2020.

Table 4 provides the full distribution of URI-Ms over archive and time. We were able to collect at least some mementos from each year between 1996–2017. Fig 8 shows the median number of resources (e.g., images, CSS/JavaScript files, and iframes) comprising composite mementos in our dataset per year. As expected, the figure indicates that web pages contain fewer resources in early years 1996–2006 as compared to recent years. Overall, there was a mean of 42 embedded resources per page, with a median of 32 resources per page.

**Table 4:** URI-Ms per archive per year.

Archive	URI-Ms	1996	97	98	99	00	01	02	03	04	05	06	07	08	09	10	11	12	13	14	15	16	17
webarchive.loc.gov	1,594	-	1	1	1	4	100	100	100	99	100	100	100	100	98	99	99	99	98	98	99	98	-
vefsafn.is	1,589	6	8	10	11	11	13	13	14	42	46	74	71	70	85	102	116	140	153	152	152	150	150
webcitation.org	1,585	-	-	-	-	-	-	-	-	-	28	89	85	70	119	156	156	157	156	155	130	127	157
arquivo.pt	1,569	30	14	14	15	15	-	-	-	-	1	1	-	163	167	166	163	162	167	165	164	162	-
web.archive.org	1,566	73	73	73	69	71	71	72	73	72	73	72	72	72	72	70	69	69	67	70	71	72	70
archive.is	1,396	11	10	9	12	10	12	14	13	18	14	20	33	25	29	28	59	12	214	214	214	213	212
wayback.archive-it.org	1,383	17	15	2	1	3	1	1	-	1	51	109	107	108	105	109	107	106	109	107	107	109	108
swap.stanford.edu	1,222	-	-	-	-	-	-	-	-	-	-	-	21	77	185	166	119	135	164	180	140	21	14
nationalarchives.gov.uk	994	-	-	-	-	-	-	1	2	25	12	50	40	97	117	106	110	104	94	83	59	54	40
europarchive.org	979	-	-	-	-	-	-	-	-	-	-	-	-	-	-	-	120	219	72	172	146	213	37
webharvest.gov	712	-	-	-	-	-	-	-	-	128	-	126	-	91	-	129	2	127	59	38	12	-	-
digar.ee	488	-	-	-	-	-	-	-	-	-	-	-	-	-	-	36	95	69	89	69	74	56	-
proni.gov.uk	469	-	-	-	-	-	-	-	-	-	-	-	-	-	-	17	94	19	75	75	78	59	52
collectionscanada.gc.ca	351	-	-	-	-	-	-	-	-	-	40	173	138	-	-	-	-	-	-	-	-	-	-
webarchive.org.uk	349	-	-	-	-	-	-	-	-	-	6	9	10	31	34	31	34	34	30	34	29	34	33
archive.bibalex.org	199	-	-	1	-	-	-	-	-	-	-	1	-	-	-	-	99	98	-	-	-	-	-
perma-archives.org	182	-	-	-	-	-	-	-	-	-	-	-	-	-	-	-	-	-	-	23	53	53	53
**Total**	**16,627**	137	121	110	109	114	197	201	202	385	371	824	677	904	1011	1215	1442	1550	1547	1635	1528	1421	926

### Step 2: Download rewritten/raw composite mementos

We downloaded the 16,627 mementos 39 times during the 422 days between November 16, 2017 and January 11, 2019. All downloaded content was stored in WARC files, with a total size of 1.46 TB. Full details of how we generated the WARC files are available in Chapter 6 of Aturban’s PhD thesis [33].

We used Squidwarc [99, 100] to download mementos. Squidwarc uses Google Chrome in headless mode (Headless Chrome) to render mementos. Headless browsing allows loading an entire web page faster as it runs without the need of the UI. Another powerful feature of Headless Chrome is its ability to execute JavaScript, which leads to the discovering of URIs to embedded resources that otherwise would not be discovered by tools that do not execute JavaScript, such as Wget [101].

Each time we downloaded a memento, we created two files, *rewritten.warc* and *raw.warc*. We replayed the memento and stored all of the resources needed to appropriately replay a composite memento, including those loaded by JavaScript, in *rewritten.warc*. To allow for content comparison between downloads, we downloaded the raw memento for each discovered resource and stored these in *raw.warc*. This is because the content of rewritten resources will often be different each time they are replayed, due to the archive modifications discussed earlier. So we do not want to use these for comparing mementos because we expect the contents to change upon each replay.

Our intuition is that we should be able to generate repeatable hashes on raw mementos, but we have found instances where this is not the case. Some archives respond to raw memento requests with an altered (or a custom) HTML base file, not the raw content. In other cases, raw mementos might be modified for security reasons (e.g., applying Email Address Obfuscation [102]) by a third-party service used by archives. So while using the raw mementos is frequently useful for establishing a baseline for comparison, there are cases where the archive will not or can not return the page as originally archived, including webcitation.org, collectionscanada.gc.ca, and archive.is. These cases will be explored further when we detail the types of changes we discovered in our study.

### Step 3: Generate aggregated hash values

Our goal is to generate a single aggregated hash value (i.e., root hash) for each downloaded composite memento. Instead of creating hashes of the entire *rewritten.warc* and *raw.warc* files as a whole, we decided to break the process down to smaller components so that when changes in the hash value occurred, it would be easier to identify the changed component. Using hashing, we wanted to be able to track the following components of a composite memento:

- *S*: The set of all resources (the base HTML file and embedded resources) that comprise a composite memento.
- *C*: The returned HTTP status code to a memento request
- *H*: The set of HTTP response headers that we do not expect to change, namely Memento-Datetime, Content-Type, Location, and all original response headers that start with X-Archive-orig-.
- *URI-M*: The URI of a memento of an original resource (e.g., https://webarchive.nationalarchives.gov.uk/20170302192821/https://cereals.ahdb.org.uk/).
- *R*: The returned HTTP entity body of a memento.

The attribute *S* is associated with each composite memento while the attributes *C*, *H*, *URI-M*, and *R* are associated with each individual memento. Ideally, when replaying a memento at different times, each attribute defined above would always have the same value. For individual resources and simple composite mementos, this can often be true. However, for pages with JavaScript and/or large |*S*|, this is often not true.

For each resource contained in *S*, we create a hash on the HTTP entity body *R*, a hash on the *URI-M*, and a hash on selected HTTP response headers *H* that are not expected to change. We include the URI-M to identify if a resource in a composite memento has redirected to a different memento. We include the Memento-Datetime HTTP header to identify if the datetime of an embedded resource has changed (could occur if the original memento was unavailable at playback time), Location HTTP header to identify if mementos were served from different locations, the Content-Type HTTP header to identify if the format of the resource changed, and original HTTP headers that begin with X-Archive-orig-. In particular, there are certain HTTP headers that we do *not* want to include, such as Date because its value changes each time the memento is accessed.

**Fig 9:**
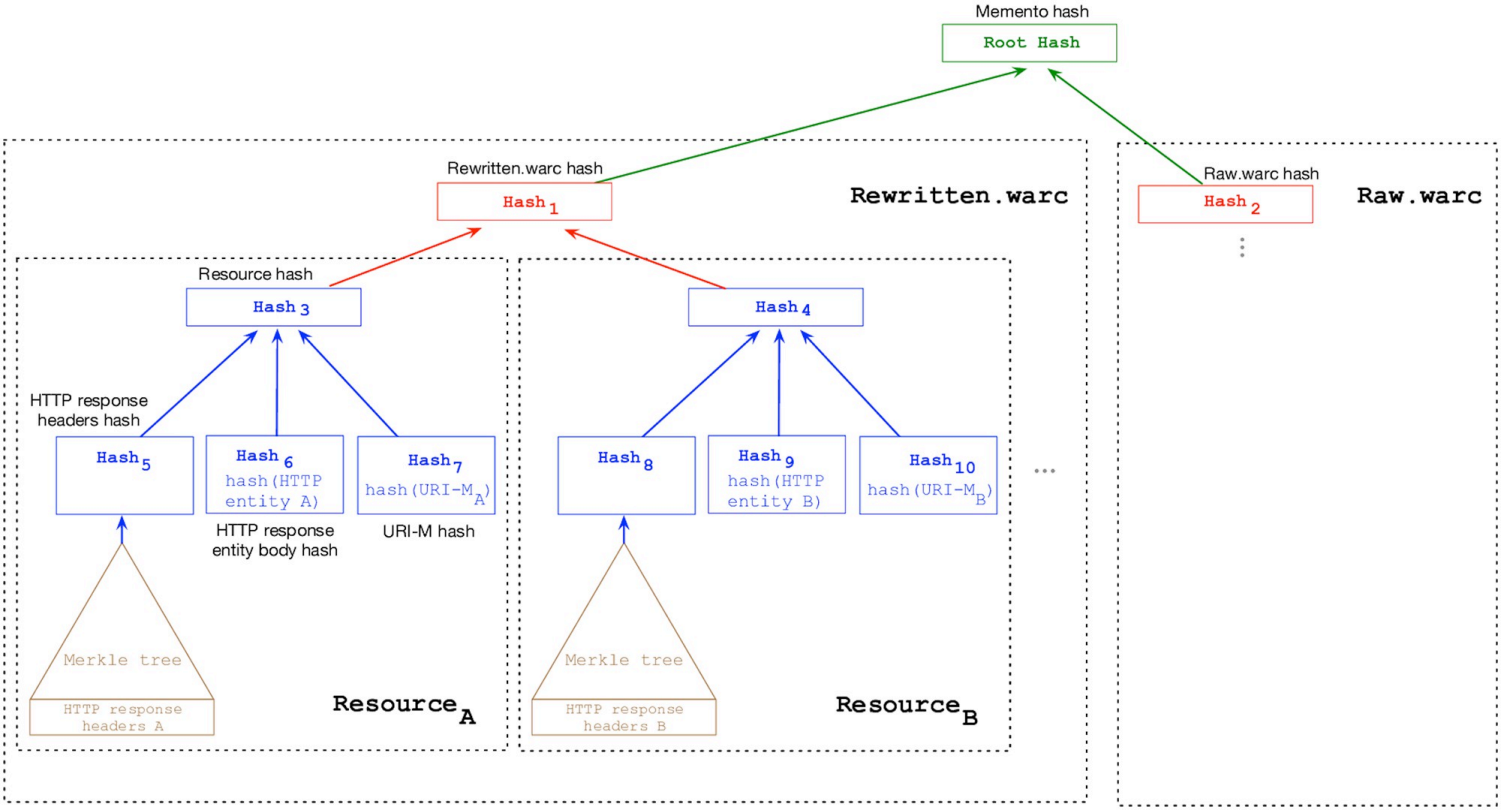
Diagram illustrating how the root hash of a memento using Merkle trees is generated. The output of a Merkle tree becomes input to another Merkle tree. The brown Merkle tree is for generating a hash on HTTP response headers of each resource. The blue Merkle tree generates an overall hash for each resource. The red Merkle tree generates a hash that represents *rewritten.warc* and another hash for *raw.warc*. The root hash is generated by the green Merkle tree. M. Aturban, A Framework for Verifying the Fixity of Archived Web Resources, PhD dissertation, 2020.

To create the root hash for each downloaded composite memento, we use *rewritten.warc* and *raw.warc* to build Merkle Trees [97]. A leaf node of a Merkle tree contains the hash of data, while a non-leaf node contains a hash of its children nodes’ hashes. As Fig 9 and Algorithm 1 show, our technique of generating the root hash of a memento is based on four Merkle trees (marked in different colors), where the output of one Merkle tree becomes input to another Merkle tree. There are multiple ways to generate a single aggregated hash, but we chose to use the Merkle tree because its design is well-suited for generating a hash of hashes and its binary hash tree structure can be used to quickly detect which resources may cause the same memento to produce different hash values.

**Algorithm 1** Generate a Root Hash Using Four Merkle Trees

**Require**: *WARC*_*rewritten*_, *WARC*_*raw*_

**Ensure**: *Hash*_*root*_

1: **function** ROOT_Hash(*WARC*_*rewritten*_, *WARC*_*raw*_)

2:  *Hash*_*rewritten*_ ← *WARC_Hash*(*WARC*_*rewritten*_)

3:  *Hash*_*raw*_ ← *WARC_Hash*(*WARC*_*raw*_)

4:  *Hash*_*root*_ ← *merkleTree_green*(*Hash*_*reritten*_, *Hash*_*raw*_)     ▹ The final root hash

5:  **return** *Hash*_*root*_

6: end function

7:

8: **function** WARC_Hash(*Resources*[])

9:  *Hash*_*resources*_ ← []

10:  *N* ← *length*(*Resources*)

11:  **for** *k* ← 1 to *N* **do**

12:   *Hdrs* ← *extractHdrs*(*Resources*_*k*_)

13:   *Hash*_*Hdrs*_ ← *merkleTree_brown*(*Hdrs*)     ▹ The hash on selected headers

14:   *Entity* ← *extractEntity*(*Resources*_*k*_)

15:   *Hash*_*Entity*_ ← *hash*256(*Entity*)

16:   *URIM* ← *extractURIM*(*Resources*_*k*_)

17:   *Hash*_*URIM*_ ← *hash*256(*URIM*)

18:   *Hash*_*rsrc*_ ← *merkleTree_blue*(*Hash*_*Hdrs*_, *Hash*_*Entity*_, *Hash*_*URIM*_)

19:             ▹ The overall resource hash

20:   *Hash*_*resources*_.*insert*(*Hash*_*rsrc*_)

21:  end for

22:  *Hash*_*WARC*_ ← *merkleTree_red*(*Hash*_*resources*_)     ▹ The overall WARC hash

23:  **return** *Hash*_*WARC*_

24: **end function**

For each resource in *rewritten.warc* and *raw.warc*, a Merkle tree (marked in brown in Fig 9) is built on the HTTP response headers of a resource. For instance, the values Hash_5_ and Hash_8_ are generated on the HTTP response headers of Resource_*A*_ and Resource_*B*_, respectively.

Next, another Merkle tree (marked in blue in Fig 9) is used to calculate the hash of each resource. The input to this Merkle tree includes (1) the resulting hash value for the HTTP response headers generated from the previous step (e.g., Hash_5_), (2) the hash of the HTTP entity body of the resource (e.g., Hash_6_), and (3) the hash of the resource’s *URI-M* (e.g., Hash_7_) After this step, we should have a single hash for each resource in *rewritten.warc* and *raw.warc* (e.g., Hash_3_ of Resource_*A*_ and Hash_4_ of Resource_*B*_ in Fig 9).

The next step is to create a Merkle tree (marked in red) that consists of all resources’ hashes in each WARC file. This step will result in only two hashes: one hash for *rewritten.warc* (e.g, Hash_1_) and the other hash for *raw.warc* (e.g, Hash_2_).

The final step is to calculate the final hash (i.e., Root Hash) using a Merkle tree (marked in green in Fig 9) where the input of this tree is the hash of *rewritten.warc* and the hash of *raw.warc*. The resulting hash can be considered as a summary of the content of a memento at a particular time.

The Merkle trees hold all possible combinations that can be involved in hash calculation, but not all of these combinations will be included in the hash calculations, because we are not always able to obtain the same information from all archives (due to differences in how mementos are replayed). For archives that provide raw mementos, everything from *raw.warc* will be included in the hash calculation, along with certain elements from *rewritten.warc*, including the hash of the HTTP status code, hash of the URI-M itself, hashes of selected HTTP headers, and hashes on entity bodies that are not text-based (e.g., images, PDF). We do not include text-based entity-bodies (e.g., HTML, CSS, JavaScript) from *rewritten.warc* because archives often inject code for archive banners, rewrite URLs, and make other modifications upon each replay (e.g., the replay date of playback shown in Fig 4). As a result, when we claim that an entity has changed, that determination was based on, for example, raw HTML or both replayed and raw JPEG, but never replayed HTML, since replayed HTML is expected to change. We also exclude elements from any live resources (i.e., non-mementos) found in *rewritten.warc*, because these are usually archive banners and other archive metadata that we expect to change on each replay. (Unexpected live resources are a point of concern, but harken back to the security issues from Lerner et al. [90] discussed in Related Work).

## Identifying types of changes on the playback of mementos

For each memento, we have 39 root hash values that were generated after each replay. We would expect to see the same content, and thus the same hash values, each time. We compared consecutive hashes: the first hash is compared with the second hash, the second hash is compared with the third hash, and so on. Each time two consecutive hash values were different, we identified one type of change causing different hashes for the same memento. In general, the change could occur on the base HTML file, embedded resources (e.g., images), or HTTP response headers.

As defined in the previous section, we use the notation *S* to represent the set of all resources in a composite memento, and for each resource in *S*, *C* represents the HTTP status code, *H* represents the selected set of HTTP response headers, and *R* represents the HTTP entity body of the resource.

### Set change

ΔS = (***S’***, *C*, *H*, *URI-M*, *R*): One or more resources in the set comprising a composite memento has changed. This may include observing new resources added, resources replaced with others, or missing resources that were previously part of the composite memento.

**Fig 10:**
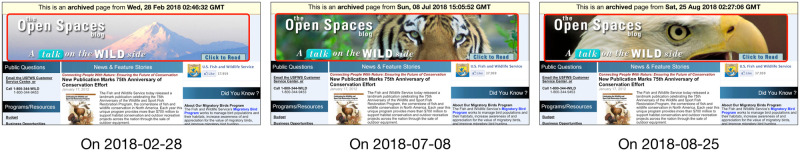
Different images shown on each replay. Replaying the memento https://www.webharvest.gov/congress112th/20130119060624/http://www.fws.gov/ at three different times produced three different images. M. Aturban, A Framework for Verifying the Fixity of Archived Web Resources, PhD dissertation, 2020.

**Fig 11:**
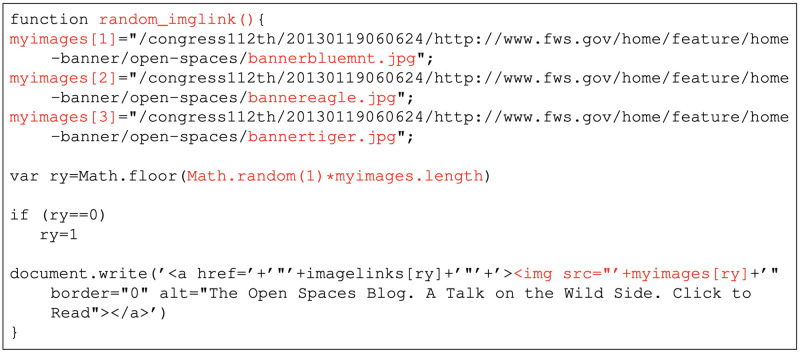
JavaScript code on https://www.webharvest.gov/congress112th/20130119060624/http://www.fws.gov/. Because of the function Math.random(), each time the JavaScript code is executed, an image will be selected randomly. M. Aturban, A Framework for Verifying the Fixity of Archived Web Resources, PhD dissertation, 2020.

Fig 10 shows an example of the *Set* change where the memento https://www.webharvest.gov/congress112th/20130119060624/http://www.fws.gov/ is replayed at three different times. Over the course of six months, we observed three different header images upon playback. Inspecting the base HTML (snippet shown in Fig 11) reveals a JavaScript function random_imglink() that randomly chooses one of *bannerbluemnt.jpg*, *bannertiger.jpg*, or *bannereagle.jpg* as the image to display. We classify this as a *Set* change because the particular embedded resource that is loaded is not determined until playback and can change each time the page is reloaded.

Although it falls outside the temporal range of our study (2017–2019), we know of another high-profile archive replay system upgrade that would impact *S*. From November 1, 2016 through sometime in Spring 2022, the Internet Archive’s Wayback Machine was unable to fully replay the top level cnn.com page archived after November 1, 2016 due to cross-origin assumptions that were invalidated by CNN’s JavaScript running at archive.org and not cnn.com [81]. When the Internet Archive upgraded their replay software sometime in Spring 2020, the replay resumed correctly. The embedded resources for the affected cnn.com pages were archived correctly, but replay failures meant those resources would not be included in *S* for a composite memento generated between late 2016 through early 2020. After the Internet Archive’s upgrade, any new replay of those affected pages would see an avalanche of new resources in *S*, which although is a more faithful replay of the archived page, would result in invalidation of any hashes computed on the composite memento.

### Status code change

ΔC = (*S*, ***C’***, *H*, *URI-M*, *R*): The HTTP status code of one or more resources comprising a composite memento has changed.

One of the reasons for changes in the HTTP status code of a resource is the technique web archives use to crawl or capture web pages. It is not uncommon to encounter a situation where the embedded resources within a composite memento have different values for Memento-Datetime. This is because archives may start serving mementos even before all of their embedded resources have been crawled. Recall that archive web crawlers place all discovered but not yet crawled URIs in a queue so that they can be processed later. Thus, it is likely that we see some archived resources with 404 Not Found at a particular time that then become 200 OK when revisited at a later time.

**Fig 12:**
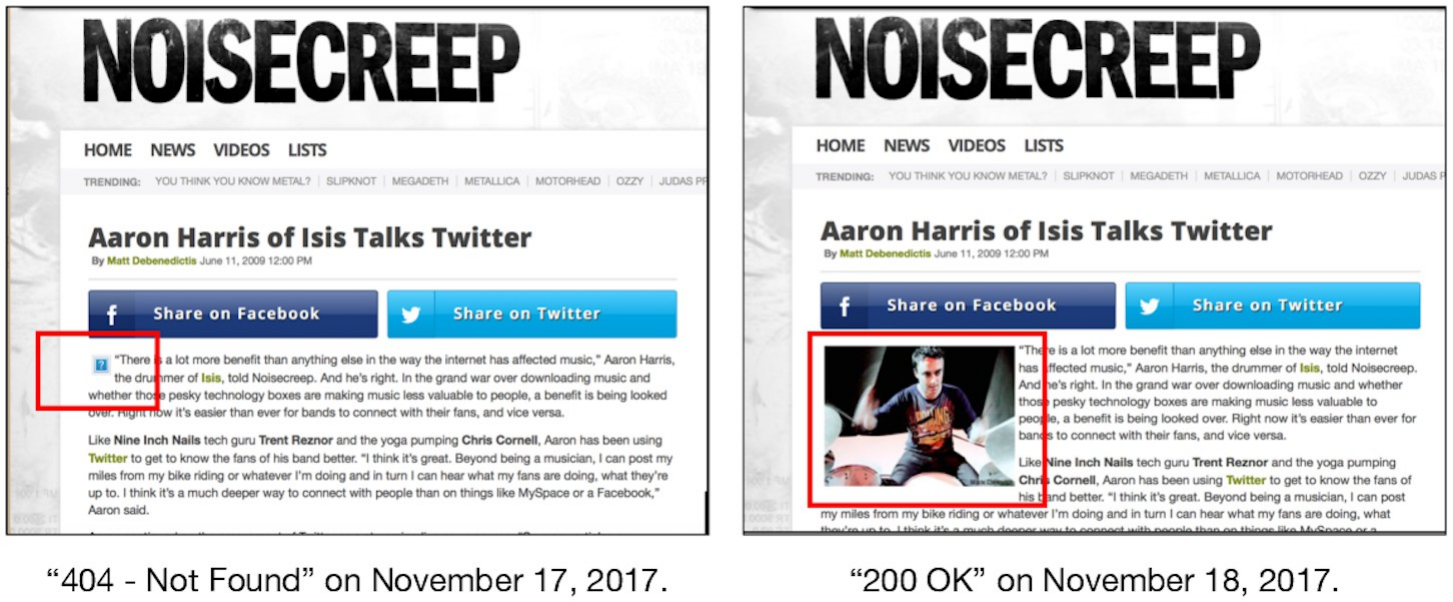
Different HTTP status codes. Replaying the memento https://web.archive.org/web/20141209193553/http://noisecreep.com/aaron-harris-of-isis-talks-twitter/ at two different times. We observe different HTTP status code of the embedded image https://web.archive.org/web/20141209193553im_/http://wac.450F.edgecastcdn.net/80450F/noisecreep.com/files/2009/06/aaron_a042209eb_200.jpg. M. Aturban, A Framework for Verifying the Fixity of Archived Web Resources, PhD dissertation, 2020.

Fig 12 shows an example of an HTTP status code change of the image https://web.archive.org/web/20141209193553im_/http://wac.450F.edgecastcdn.net/80450F/noisecreep.com/files/2009/06/aaron_a042209eb_200.jpg which is embedded in the memento https://web.archive.org/web/20141209193553/http://noisecreep.com/aaron-harris-of-isis-talks-twitter/. The HTTP status code of the image was 404 the first time it was requested on November 17, 2017 (as illustrated in the red square on the left in Fig 12). When requesting the same memento for the second time on November 18, 2017, the HTTP status code of the same embedded image had become 200. The Internet Archive tries to archive embedded resources that are requested but not yet archived. In this example, the November 17 request for the missing image triggered a 302 Redirect to a special URI, https://web.archive.org/save/_embed/http://wac.450F.edgecastcdn.net/80450F/noisecreep.com/files/2009/06/aaron_a042209eb_200.jpg. The browser rendering the composite memento would then follow the redirect and issue a request for this URI. This resource (i.e., web.archive.org/save/_embed/<URI-R>) triggered a service in the archive to capture the image from the live web. Then the archive was able to respond to the November 18 request with 302 Redirect to the URI-M of the newly archived image https://web.archive.org/web/20171118103250/http://wac.450F.edgecastcdn.net/80450F/noisecreep.com/files/2009/06/aaron_a042209eb_200.jpg.

This example indicates that just by requesting mementos, we may actually change the archive, since these requests trigger a service in the archive to capture resources that have not yet been archived. There is a trade-off between capturing resources at the request time (as in the example of the image above) and simply returning 404 Not Found. From the regular viewer’s perspective, the archive takes the right action by adding a missing resource in a composite memento by retrieving it from the live web (aka “patching” the archive) [103]. On the other hand, for a user interested in computing fixity information, this action affects generating repeatable hashes: on each replay, the composite memento may *improve* via fewer missing resources, but the hashes for each replay will not match.

In addition, the HTTP status code may change because of transient errors. Archives frequently respond with a 5xx HTTP status code if unable to serve resources at certain times. Also, the HTTP status code change can occur when archives apply updates to their replay services. For example, after deploying a new version of PyWB, the archive webarchive.org.uk started responding with 307 Temporary Redirect to requests that previously returned 302 Found. This server upgrade is opaque to interactive users (browsers silently handle all HTTP redirects), but could impact hashing technique that relied on *C*.

It is also possible that different archival redirects for the same HTTP request eventually return different HTTP status codes. On December 07, 2017, the image http://webarchive.loc.gov/all/20001225075832/http://www.senate.gov/resources/sidebar_top.gif. redirected to a URI-M with the Memento-Datetime December 10, 2001 07:18:11 GMT with the HTTP status code 200 OK. When the same image was requested on December 14, 2017, it redirected to a different URI-M with the Memento-Datetime December 25, 2000 19:58:25 GMT with the HTTP status code 415 Unsupported Media Type.

### Header change

ΔH = (*S*, *C*, ***H’***, *URI-M*, *R*): An HTTP response header that we do not expect to change has changed. As we mentioned, some HTTP response headers are not expected to change, including Memento-Datetime, Content-Type, and the original headers that begin with X-Archive-orig-. However, sometimes one or more of these headers do change, often as a result of a change in the configuration of the web archive itself. For example, the response header Content-Type changed multiple times for: https://web.archive.org/web/20071111211818/http://images.sohu.com:80/chat_online/market/sohu/140140-1.html On December 30, 2017, the value of the response header Content-Type was text/html; charset = utf-8. The value changed to text/html; charset = gb2312 on January 31, 2018.

### URI-M change

ΔURI-M = (*S*, *C*, *H*, ***URI-M’***, *R*): One or more resources in the set comprising a composite memento has a lexicographical change in its URI-M.

**Fig 13:**
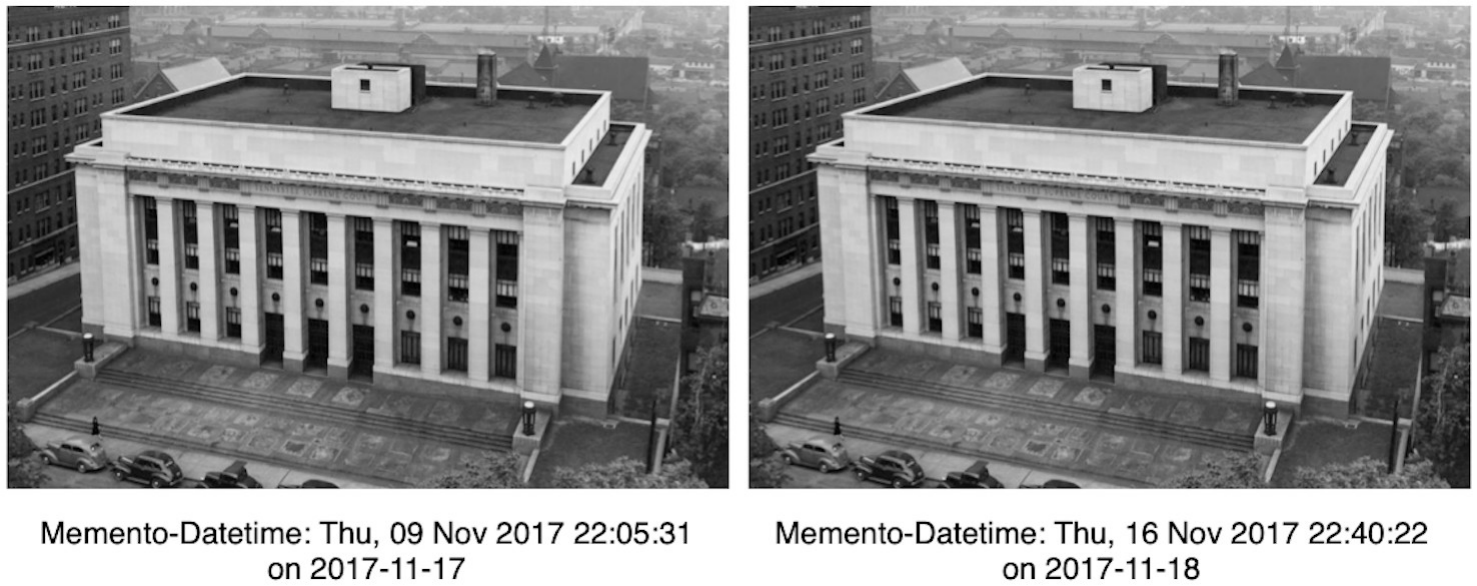
Different URI-M, but identical HTTP entities. Requesting the image https://web.archive.org/web/20171114170029im_/https://sos.tn.gov/sites/default/files/styles/large/public/15259.jpg?itok=BgNjlAZj which is embedded in the memento https://web.archive.org/web/20171114170029/https://sos.tn.gov/tsla at two different times. Each time, it redirects to a different URI-M with different Memento-Datetime, but the returned HTTP entities are identical.

Frequently this type of change is observed when the web archive redirects to a resource captured at a different Memento-Datetime, which results in a lexicographically different URI-M. For example, Fig 13 illustrates the scenario where the same HTTP request for an image was sent at two different times. Each time the archive returned a 302 Redirect to a different URI-M, but the HTTP entities of these two responses were identical. Embedded resources, such as images and style sheets, often change slowly, if at all, so frequently versions archived at different Memento-Datetimes are functionally interchangable even if the URI-M is lexicographically different.

In general, we identify two URI-Ms as “different” if either their URI-Rs or Memento-Datetimes are different. However, there are other cases where two lexicographically different URI-Ms canonicalize to the same value. For example, we requested the same URI-M http://perma-archives.org/warc/20170303200708/http://id.loc.gov/ at two different times on March 27, 2018 and July 08, 2018. The only difference between the two responses is that the archive perma.cc started serving over HTTPS (instead of HTTP) on or around July 08, 2018. In such cases, without canonicalizing URI-Ms (e.g., HTTP = HTTPS), they will produce different hashes.

Archives may serve requested mementos in iframes. For example, webarchive.org.uk began supporting the option mp_ for loading the archived content into an iframe. Therefore, any new requests for mementos from this archive will result in 302 redirect to a URI-M that has mp_ after the timestamp.

The *URI-M* change may also be referred to as TimeMap change, where archives respond, at different times, with various TimeMaps of the same *URI-R*. The TimeMap inconsistency occurs for reasons including deduplication and redaction techniques of mementos, archival restructuring, and transient errors [104]. The TimeMap change causes requests of the same memento to redirect to different URI-Ms.

### Representation change

ΔR = (*S*, *C*, *H*, *URI-M*, ***R’***): The returned HTTP entity body of one or more resources comprising a composite memento has changed.

We saw an example of the *Representation* change when we requested the raw content of the same memento multiple times from perma.cc. We were expecting to always be presented with same raw content, but we noticed a different HTTP entity returned each time. The actual change in the returned content was not caused by the archive, but by Cloudflare, a third-party service used by the archive. This service modifies the HTML content being returned to the client by applying Email Address Obfuscation [102] to hide any email address included in the content and help to prevent spam.

**Fig 14:**
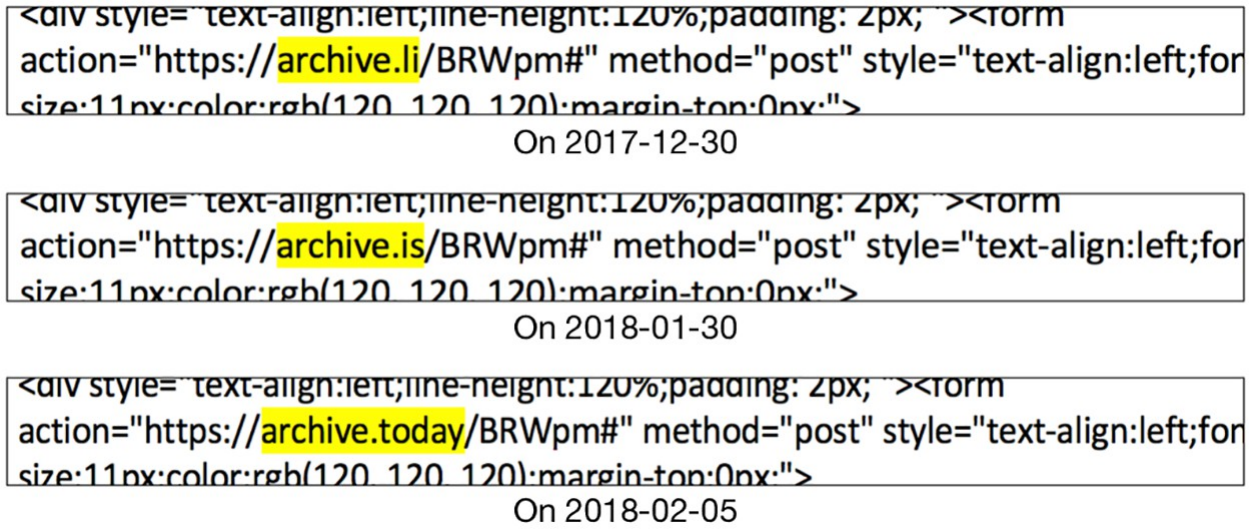
archive.is refers to itself differently in the HTML upon multiple downloads. Downloading the ZIP file http://archive.is/download/BRWpm.zip of the memento http://archive.is/BRWpm at three different times. Each time the archive refers to itself differently in the index.html in the ZIP file. M. Aturban, A Framework for Verifying the Fixity of Archived Web Resources, PhD dissertation, 2020.

Fig 14 shows another example of HTTP entity change. Recall that archive.is does not serve raw mementos, but provides a ZIP file with the rewritten content (though without banners or other headers that would change on each replay). At replay time, this archive inconsistently refers to itself using different TLDs, such as .li, .is, .ph, and .today. In particular, this change occurs in the content of the index.html in the returned ZIP file, as links to embedded resources that are also archived may have different domains depending on when the memento was replayed.

**Fig 15:**
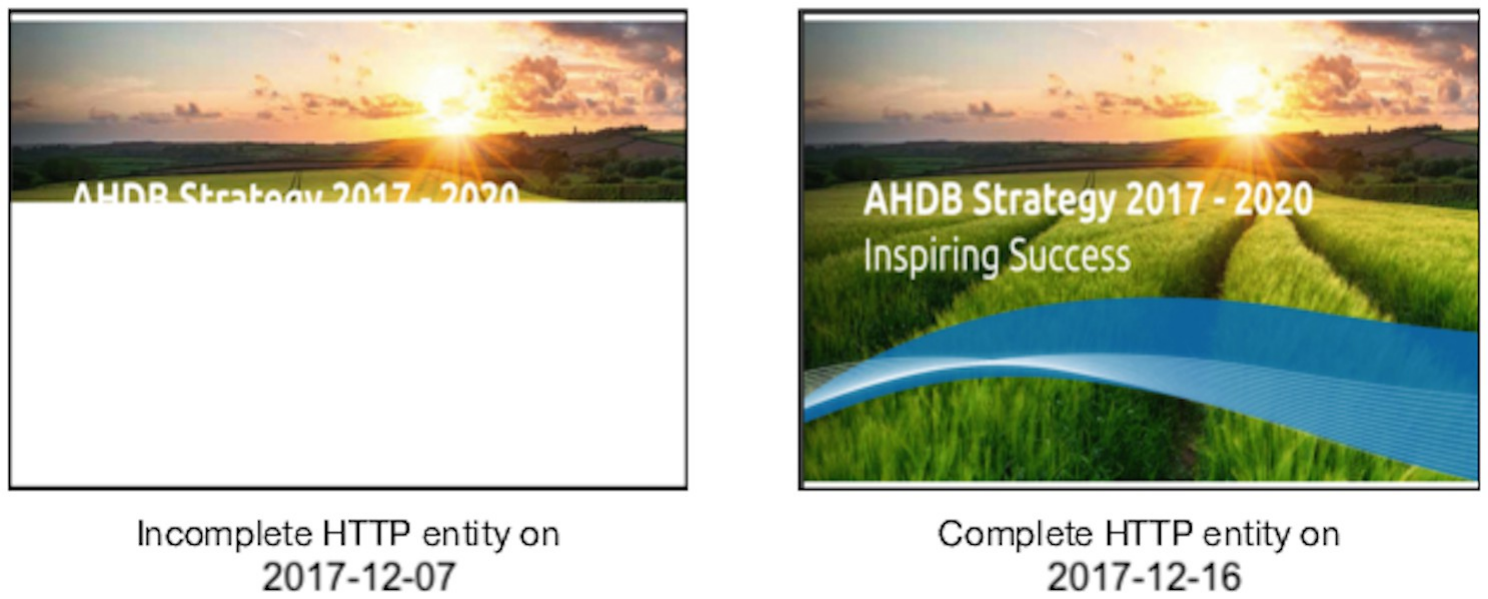
HTTP entity change. Requesting the image http://webarchive.nationalarchives.gov.uk/20170303010736id_/https://cereals.ahdb.org.uk/media/1157842/corporate-strategy-1.jpg which is embedded in the memento http://webarchive.nationalarchives.gov.uk/20170303010736id_/https://cereals.ahdb.org.uk/ at two different times. We noticed HTTP entity change because of a transient error. M. Aturban, A Framework for Verifying the Fixity of Archived Web Resources, PhD dissertation, 2020.

Furthermore, we may observe HTTP entity changes because of transient errors as shown in Fig 15. The image https://webarchive.nationalarchives.gov.uk/20170303010736id_/https://cereals.ahdb.org.uk/media/1157842/corporate-strategy-1.jpg which is embedded in the memento https://webarchive.nationalarchives.gov.uk/20170303010736id_/https://cereals.ahdb.org.uk/ was requested at two different times. The HTTP entity of the image was transferred incompletely the first time it was requested, while the entity was complete when requested for the second time.

**Fig 16:**
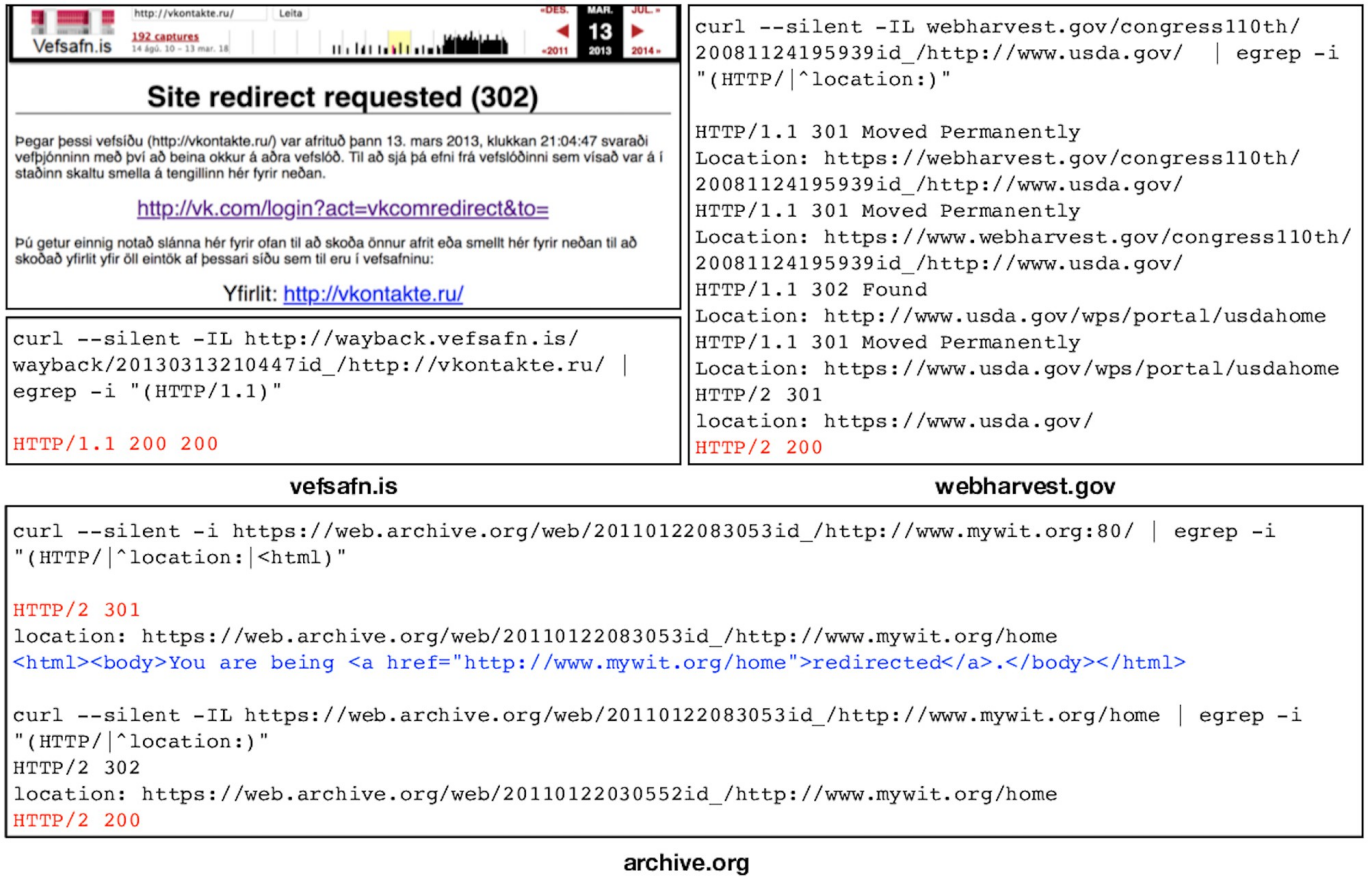
Three archives react differently to requests for raw mementos. The archive vesafn.is returns a custom HTML page with 200 OK which might cause different hashes. The archive webharvest.gov issues 302 Redirect to the live web, while archive.org returns 302 Redirect (with the original, raw HTML page—marked in blue) to the closest raw memento that satisfies the request. The way that webharvest.gov and archive.org react to requests for raw mementos does not affect the hash calculation. M. Aturban, A Framework for Verifying the Fixity of Archived Web Resources, PhD dissertation, 2020.

As demonstrated in Fig 16, we have also noticed different behavior by archives in response to requests for raw mementos when the original resource had an HTTP status code of 302 Redirect (i.e., archived 302):

- **vefsafn.is**: The Icelandic Web Archive vefsafn.is returns a custom HTML page with 200 OK, illustrated in Fig 16 (**vefsafn.is**). The returned page is not the raw version of the memento, but contains links pointing to the closest memento that satisfies the request. Such behavior might be applied by the archive to prevent redirects to the live web, but the rewritten content affects the hash calculation, resulting in different hash values.
- **webharvest.gov**: Fig 16 (**webharvest.gov**) shows why vefsafn.is inserts a custom HTML page in place of the redirect: by strictly not modifying any of the HTTP response including the Location HTTP response header, replaying the original redirect leads the client out of the web archive and to the live web, even if the target resource is in fact archived.
- **archive.org**: As shown in Fig 16 (**archive.org**), the Internet Archive correctly replays the raw entity (marked in blue), while rewriting the Location HTTP response header. This keeps the user in the web archive while returning the original entity.

The custom HTML page returned by vefsafn.is might prevent generating repeatable hashes because the returned HTML page has content that is expected to change (e.g., the banner) as the web archive software is updated. Because we do not consider any resources retrieved from the live web in hash calculation, the 302 Redirects to the live web by webharvest.gov do not affect generating repeatable hashes. We exclude any redirects to the live web in hash generation because live web resources are not expected to remain unchanged. By that same line of thought, it would be possible to implement a stricter policy that considers any redirects to the live web as a change. The technique that archive.org uses to respond to raw requests does not affect hash calculation because the archive returns the raw HTML entity and only rewrites the Location HTTP response header.

### URI-M and representation change

**Fig 17:**
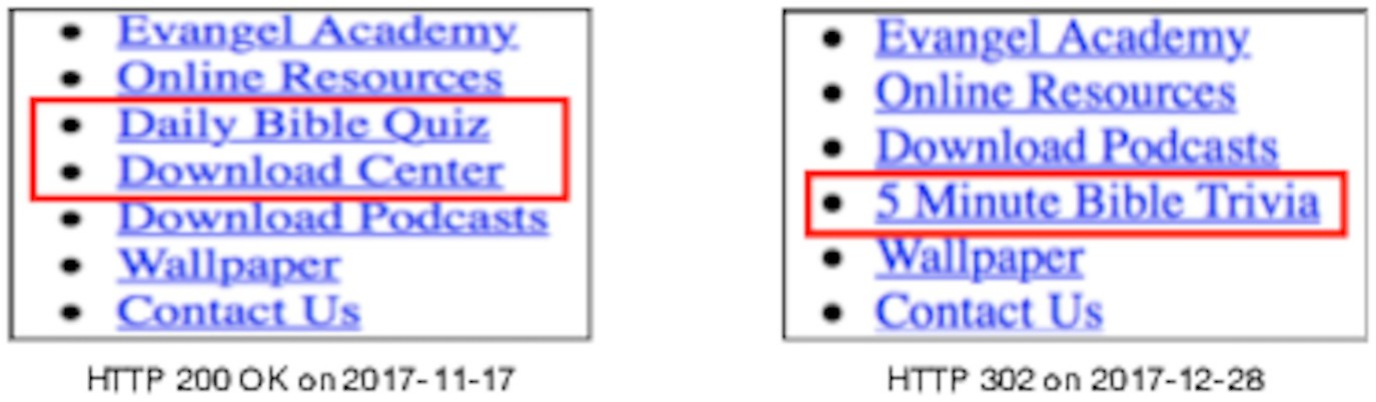
Redirect to a memento that has different HTTP entity. Requesting the base HTML file https://web.archive.org/web/20080828005922id_/http://www.evangelcogdayton.org/ at two different times. The second request on December 28, 2017 redirects to a memento that has a different HTTP entity. M. Aturban, A Framework for Verifying the Fixity of Archived Web Resources, PhD dissertation, 2020.

ΔURI-M, ΔR = (*S*, *C*, *H*, ***URI-M’***, ***R’***): The *URI-M* and *Representation* change occurs when one or more resources in the set comprising a composite memento has both a lexicographically different URI-M as well as change in the HTTP entity body. This commonly happens when the web archive redirects a request to a resource with a different Memento-Datetime, thus resulting in a different URI-M, and that resource has changed relative to the prior request. For example, Fig 17 shows that requesting the same base HTML file https://web.archive.org/web/20080828005922id_/http://www.evangelcogdayton.org/ at two different times results in two different HTTP entities. The first HTTP request is made on November 17, 2017, and the archive responded with 200 OK as shown in Fig 17. We requested the same memento (URI-M) on December 28, 2017. The memento with the Memento-Datetime August 28, 2008 00:59:22 GMT redirects to the URI-M with the Memento-Datetime November 02, 2009 15:16:09 GMT https://web.archive.org/web/20090211151609id_/http://www.evangelcogdayton.org:80. which has a different HTTP entity.

**Fig 18:**
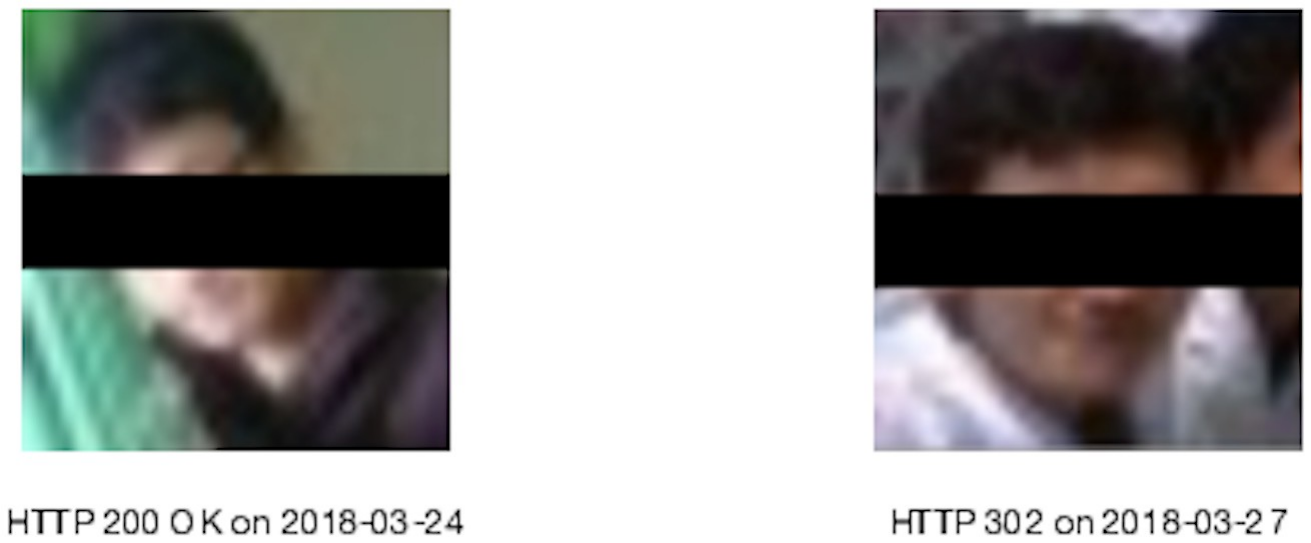
Redirect to a memento with the same URI-R, but different HTTP entity. Requesting the image https://web.archive.org/web/20110116134258id_/http://1.gravatar.com/avatar/117a6cc4203b951f11fc43f946106657?s=33&d=http%3A%2F%2F1.gravatar.com%2Favatar%2Fad516503a11cd5ca435acc9bb6523536%3Fs%3D33&r=G, which is embedded in the memento https://web.archive.org/web/20110114074814/http://www.copyblogger.com:80/popular-blogger/, at two different times. The first HTTP request returns 200 OK, but the second request redirects to a URI-M (with the Memento-Datetime January 21, 2012 09:05:32 GMT) that has the same URI-R but a different HTTP entity. M. Aturban, A Framework for Verifying the Fixity of Archived Web Resources, PhD dissertation, 2020.

**Fig 19:**
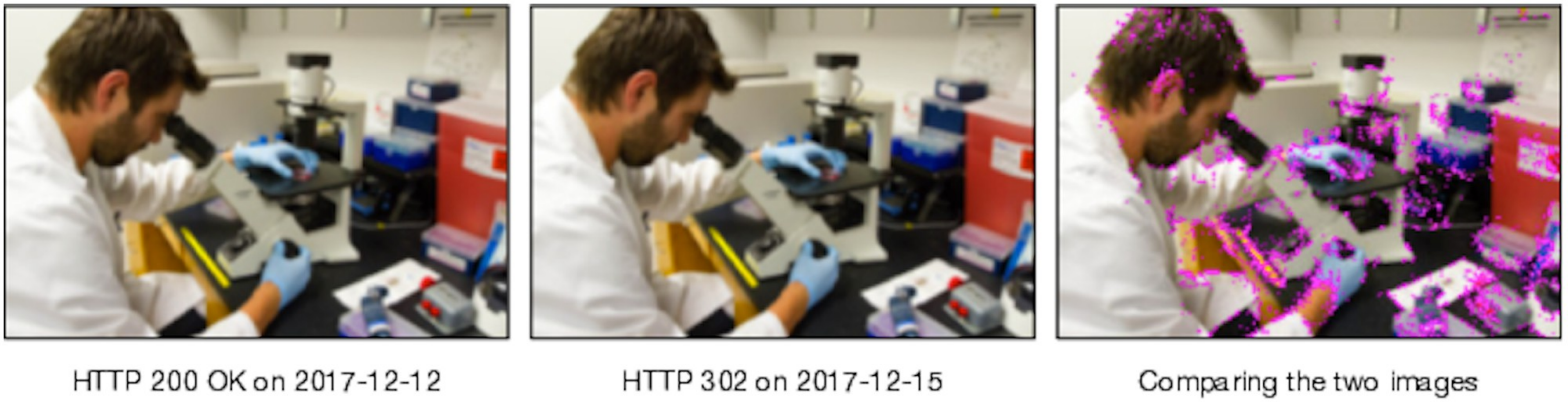
Different HTTP entity, but image looks the same. Requesting the image https://perma-archives.org/warc/20170101182814id_/http://umich.edu/includes/image/type/gallery/id/113/name/ResearchDIL-19Aug14_DM%28136%29.jpg/width/152/height/152/mode/minfit, which is embedded in the memento https://perma-archives.org/warc/20170101182813/http://umich.edu/ at two different times. The first HTTP request returns 200 OK, while the second HTTP request of the image redirects to a URI-M (with the Memento-Datetime June 19, 2017 14:54:58 GMT) which has a different HTTP entity that looks exactly the same. The two images were compared using Resemble [105] (mismatched pixels are marked in pink). M. Aturban, A Framework for Verifying the Fixity of Archived Web Resources, PhD dissertation, 2020.

The 302 Redirects are issued based on what resources are available or can be served by the archive at the time of the HTTP requests (i.e., this is also called a TimeMap change as explained in the previous section). Figs 18 and 19 show other examples where changes in URI-Ms (through 302 Redirects) resulted in different HTTP entities. The HTTP entity change in Fig 17 occurs in the base HTML file, while changes in the HTTP entities in Figs 18 and 19 affect embedded images within composite mementos. Furthermore, the different images in Fig 19 look the same, but we were able to identify differences between the two images using the image comparison tool Resemble [105].

### Timeout/Not resolved

The *Timeout/Not resolved* change occurs when one or more HTTP requests of resources in the set comprising a composite memento has a connection timeout error. In general, this type of change refers to a situation where there is no HTTP response returned from the server, not even an HTTP status code.

## Results

### Almost 90% of the mementos have at least two different hashes

**Fig 20:**
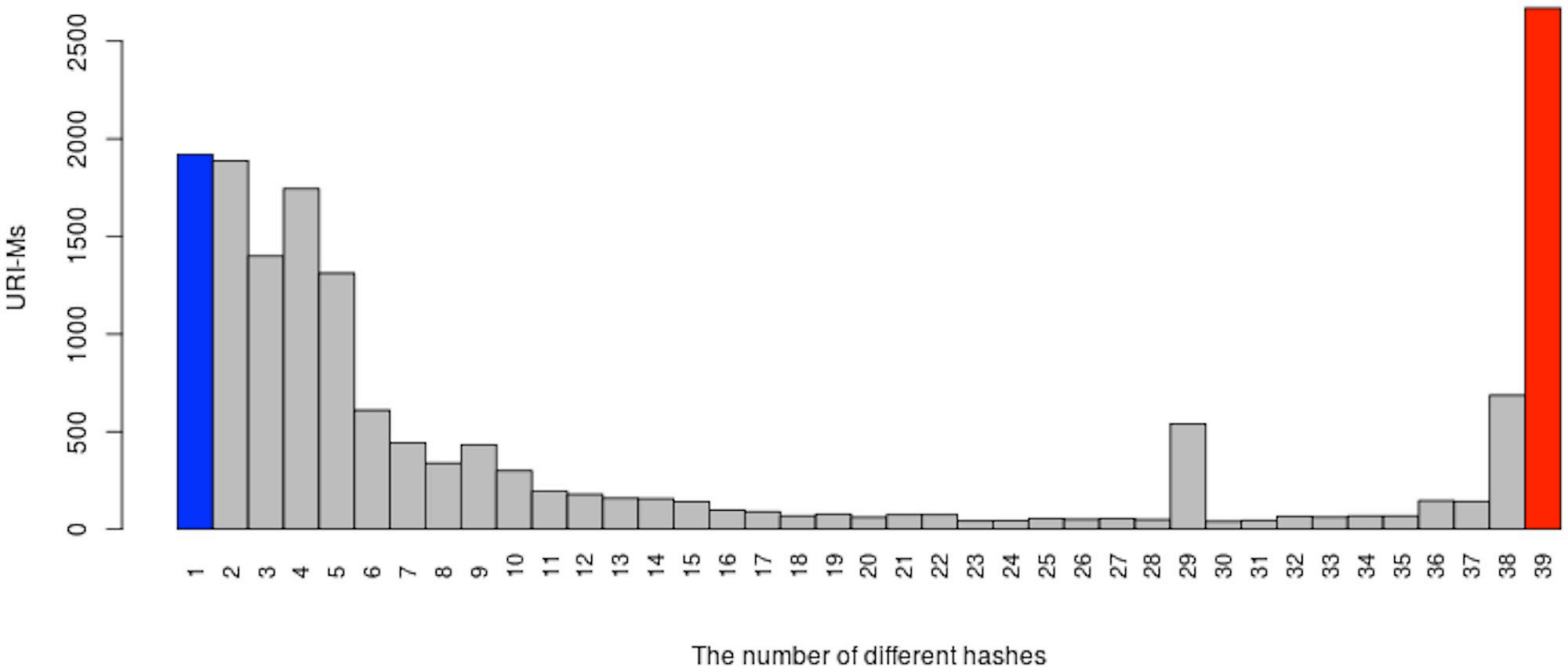
Distribution of the number of distinct hash values over all 16,627 mementos. The blue bar represents mementos with a single hash value for all downloads (1,920 mementos, 11.55%), and the red bar represents mementos with a different hash value on each download (2,670 mementos, 16.06%). M. Aturban, A Framework for Verifying the Fixity of Archived Web Resources, PhD dissertation, 2020.

We calculated the hash values for 39 downloads of each of our 16,627 mementos. Table 5 shows the number of mementos per archive that have at least two different hashes, always produced the same hash, and always produced a different hash. Almost 90% of the mementos (14,707, 88.45%, or approximately seven out of eight) have at least two different hashes, including all mementos from seven of the archives. This is even after our efforts to ensure that URL rewriting and archive-injected resources would not influence the computed hash. Fig 20 shows the number of distinct hash values per memento over all archives. The blue bar on the left indicates when there is only a single hash value (same as “always produced the same hash” in Table 5). Our intuition was this would be the most common case, but this occurs for 11.55% (1,920) of our mementos. Of the 1,920 mementos that always produced the same hash, the highest number (587) was from webcitation.org (Table 5), which archives little to no JavaScript, resulting in poor replay of archived web pages but an increased chance of a persistent hash value. Even more alarming is the red bar on the right, which indicates 39 different hash values, meaning a different hash value was computed every time the memento was replayed (same as “always produced a different hash” in Table 5). This occurred for over 16% (2,670) of our mementos.

**Table 5:** Mementos per archive that produced at least two different hashes, the same hash, or always different hashes.

Archive	URI-Ms	with at least two different hashes (%)	always produced the same hash (%)	always produced a different hash (%)
webarchive.loc.gov	1,594	1,241 (77.85)	353 (22.14)	139 (8.72)
wayback.vefsafn.is	1,589	1,138 (71.62)	451 (28.38)	99 (6.23)
webcitation.org	1,585	988 (62.97)	587 (37.03)	179 (11.29)
arquivo.pt	1,569	1,563 (99.62)	6 (0.38)	857 (54.62)
web.archive.org	1,566	1,447 (92.40)	119 (7.60)	288 (18.39)
archive.is	1,396	1,379 (98.78)	17 (1.22)	0 (0)
wayback.archive-it.org	1,383	1,383 (100)	0 (0)	216 (15.62)
swap.stanford.edu	1,222	1,021 (83.55)	201 (16.45)	308 (25.20)
nationalarchives.gov.uk	994	986 (99.20)	8 (0.8)	243 (24.45)
europarchive.org	979	979 (100)	0 (0)	0 (0)
webharvest.gov	712	712 (100)	0 (0)	123 (17.27)
veebiarhiiv.digar.ee	488	310 (63.52)	178 (36.48)	94 (19.26)
webarchive.proni.gov.uk	469	469 (100)	0 (0)	119 (25.37)
collectionscanada.gc.ca	351	351 (100)	0 (0)	0 (0)
webarchive.org.uk	349	349 (100)	0 (0)	5 (1.43)
archive.bibalex.org	199	199 (100)	0 (0)	0 (0)
perma-archives.org	182	182 (100)	0 (0)	0 (0)
**Total**	**16,627**	**14,707 (88.45)**	**1,920 (11.55)**	**2,670 (16.06)**

**Fig 21:**
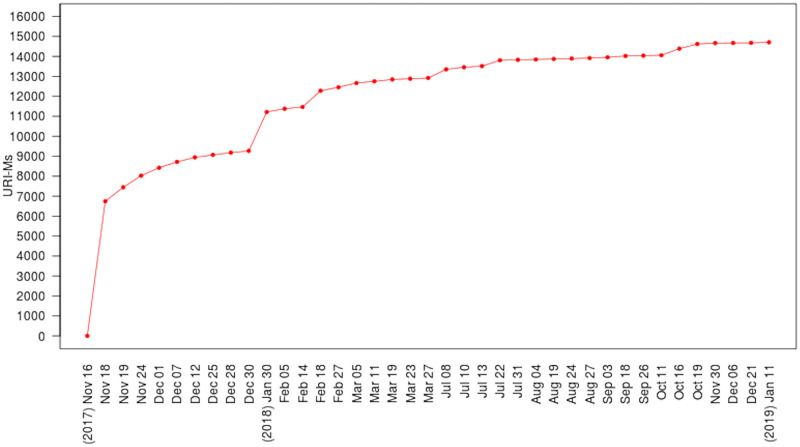
Number of mementos that have at least two different hashes increases over time. This shows that the chance of getting different hashes for the same memento increases over time. M. Aturban, A Framework for Verifying the Fixity of Archived Web Resources, PhD dissertation, 2020.

In other words, the conventional hashing approach to determine fixity in digital preservation settings works properly only for about one out of every eight mementos in our sample. Fig 21 illustrates how the pool of mementos that had at least two distinct hashes increased over time. About 40% of the mementos produced different hashes after download 2 on November 18, 2017. Then, after 37 more downloads (within 420 days), the cumulative percentage had increased to 88.45% by January 11, 2019. This illustrates our observation that the chance of getting different hashes for the same memento increases over time. We will examine some reasons for these hash differences in the next section.

### Quantifying the types of changes

To understand the types of changes that might cause different hash values for the same memento, we compared consecutive hashes for each memento. Each time any two consecutive hash values are different, we identify at most one type of change causing the different hashes, even though multiple categories might apply. We look for changes in the following order: *Set*, *Status*, *URI-M* or *Headers* (neither affects HTTP entity body), *Representation*, and *URI-M and Representation*. For example, if we detected that the set of resources comprising a memento at time *t*_0_ varies from the set at *t*_1_, then this change is marked as *Set* and other categories of changes are not considered, because if sets are different, it implies that hashes will be also different. Similarly, if sets are identical, but there are some differences in the resources’ HTTP status codes, then we assign the type of change *Status*, and no other types are considered.

**Fig 22:**
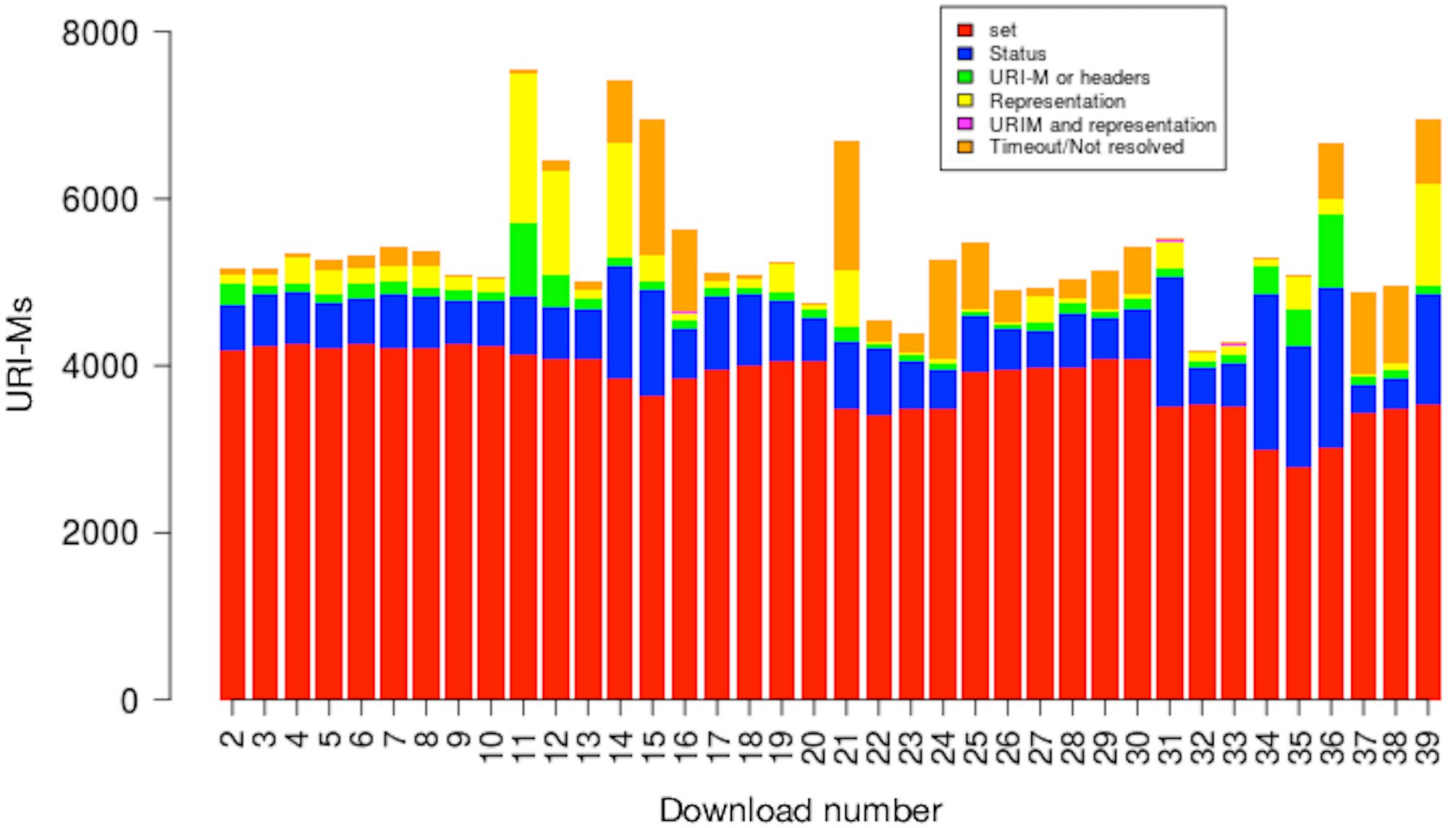
Types of changes affecting all mementos for each download. There were a total of 16,627 mementos replayed in each download. M. Aturban, A Framework for Verifying the Fixity of Archived Web Resources, PhD dissertation, 2020.

Fig 22 shows the types of changes affecting all mementos in each download. On average only about one-third of the mementos have changes when comparing consecutive downloads. Download 11, which was the first download in 2018, has the most mementos with changes, 7,557 (about 45% of the mementos). We can see that the *Set* change is the most prevalent, but recall that if we detect this change, we stop looking for other types of changes. A *Set* change means that we may have observed new resources, a resource was replaced with another, or a previously seen resource has gone missing. After *Set*, the next most frequent types of changes are *Representation*, *Timeout*, and *Status*.

**Fig 23:**
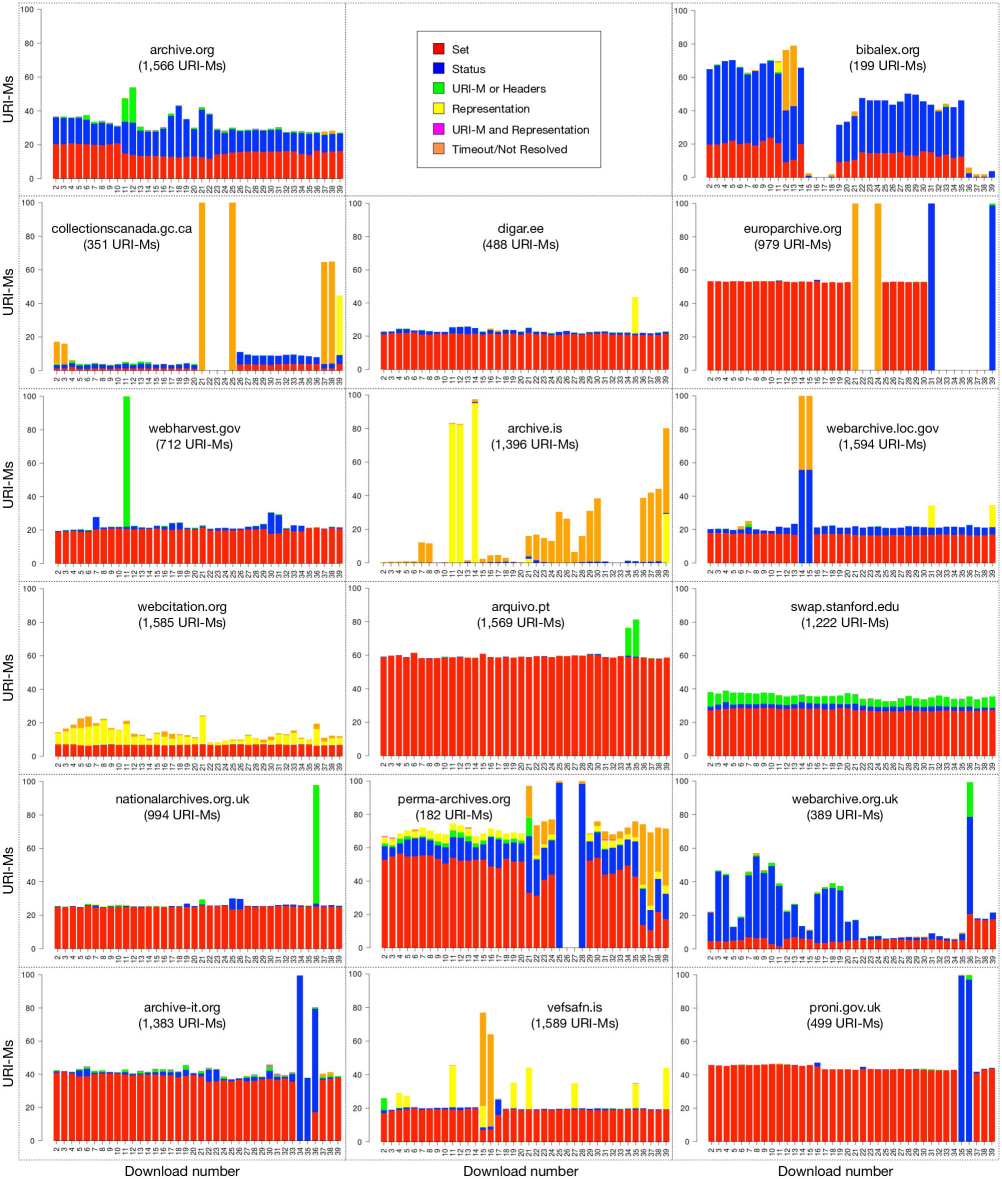
Percentage of mementos with each type of change in each download by archive. M. Aturban, A Framework for Verifying the Fixity of Archived Web Resources, PhD dissertation, 2020.

To drill-down a bit, we show the percentage of mementos with each type of change in each download for each archive in Fig 23. As expected, a large percentage of mementos from each archive are producing different hashes because of the *Set* change. This is mainly caused by dynamic resources generated after executing JavaScript, such as the three different images loaded by JavaScript on the www.fws.gov memento shown in Fig 10. The *Representation* change is detected mainly in just a few archives. We mentioned earlier that for archive.is we use a rewritten version to create the hash since the original raw source is not available and that in rewritten links in index.html, the archive often refers to itself with different domains. For instance, in download 11 archive.is used archive.li, in download 12 it used archive.is, and in download 14 it used archive.today. This caused the comparisons of downloads 10 vs. 11, 11 vs. 12, and 13 vs. 14 to show the *Representation* change because of the different rewritten hostnames for archived links. The archives vefsafn.is, webcitation.org, and perma-archives.org are also tagged with the *Representation* change. For vefsafn.is it is because the archive returns a rewritten page with HTTP 200 for requests for raw mementos, as mentioned earlier. The webcitation.org archive does not provide raw content, so our hash calculations are made on the rewritten content, which results in *Representation* changes in every download. The perma-archives.org archive uses a third-party service (Cloudflare) to prevent spam, which modifies the content being returned to the user to obfuscate email addresses. Thus, every memento that contains an email address in the HTML will have different content on each replay.

### Archive-level changes

**Fig 24:**
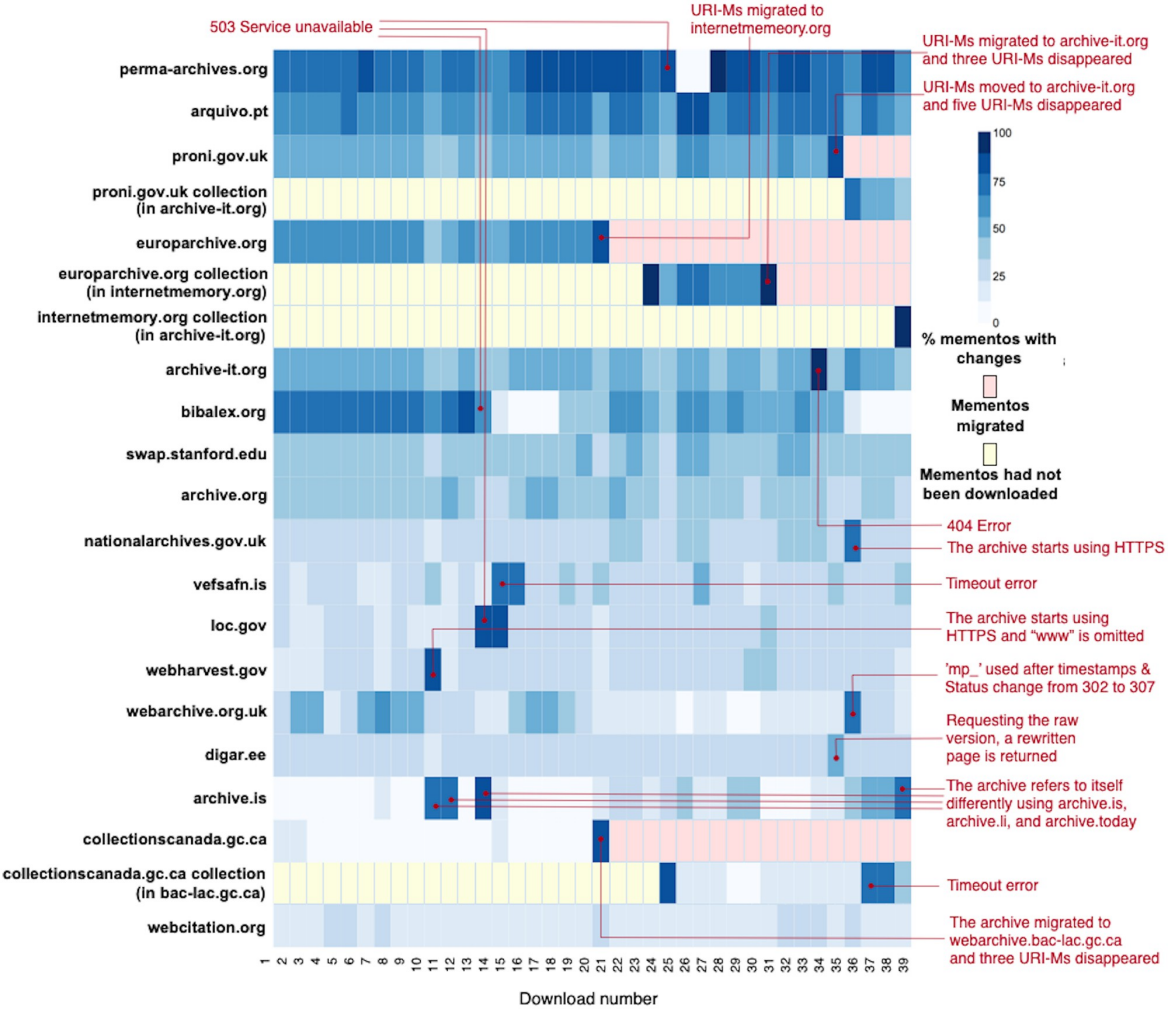
Percentage of mementos in each archive showing changes from the previous download. Light blue = fewer mementos with changes compared to a previous download, dark blue = more of the mementos have one or more changes. M. Aturban, A Framework for Verifying the Fixity of Archived Web Resources, PhD dissertation, 2020.

Fig 24 shows how each web archive behaves based on comparing consecutive downloads of mementos. Each cell represents the percentage of mementos with changes at a particular download compared with the previous download (white indicates no change, while dark blue indicates a large percentage of mementos have changed). This heatmap can be used to identify points in time where major changes occur.

For example, the mementos of the NLI collection in europarchive.org became unreachable in download 21. We discovered the new location starting from download 24 (i.e., internetmemory.org presented in the next row) [106]. Then, these NLI mementos in internetmemory.org became inaccessible, returning 403 Error, at download 31. We manually discovered the new location archive-it.org of these migrated mementos at download 39. As another example, we noticed that the performance of the webarchive.org.uk archive before download 20 is totally different from the behavior after download 20. From the WARC files, we found that the archive had upgraded the replay service to a new version of PyWB. (We discovered the change because the archive began supporting the mp_ option for loading the archived content into an iframe.)

In general, we should be cautious in interpreting the heatmap because multiple consecutive cells with similar colors do not always imply good stable behavior. For example, there are some periods where all mementos from particular archives were not available, like mementos from perma-archives.org in downloads 25, 26, and 27 (i.e., returning 500 Internal Server Error between July 31, 2018 and August 19, 2018), so the white cells indicate no changes in mementos, but they were actually unavailable during those three downloads. We added annotations to the heatmap to explain some of the major archive-level changes.

### Changes on each download

**Fig 25:**
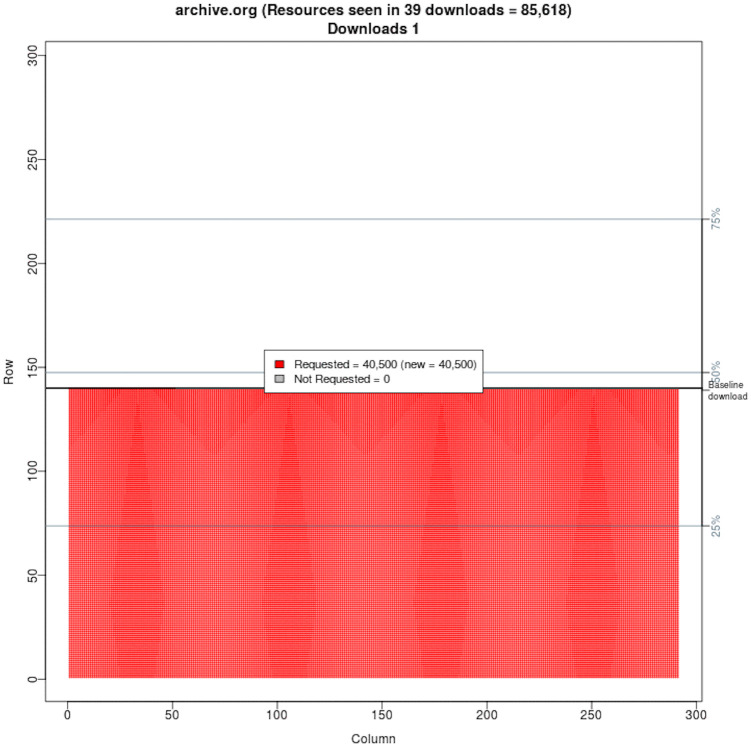
Hash calculations for the resources from 1,566 composite mementos from the Internet Archive in download 1. Each point = hash(HTTP response headers, HTTP entity body, HTTP status code, URI-M). M. Aturban, A Framework for Verifying the Fixity of Archived Web Resources, PhD dissertation, 2020.

**Fig 26:**
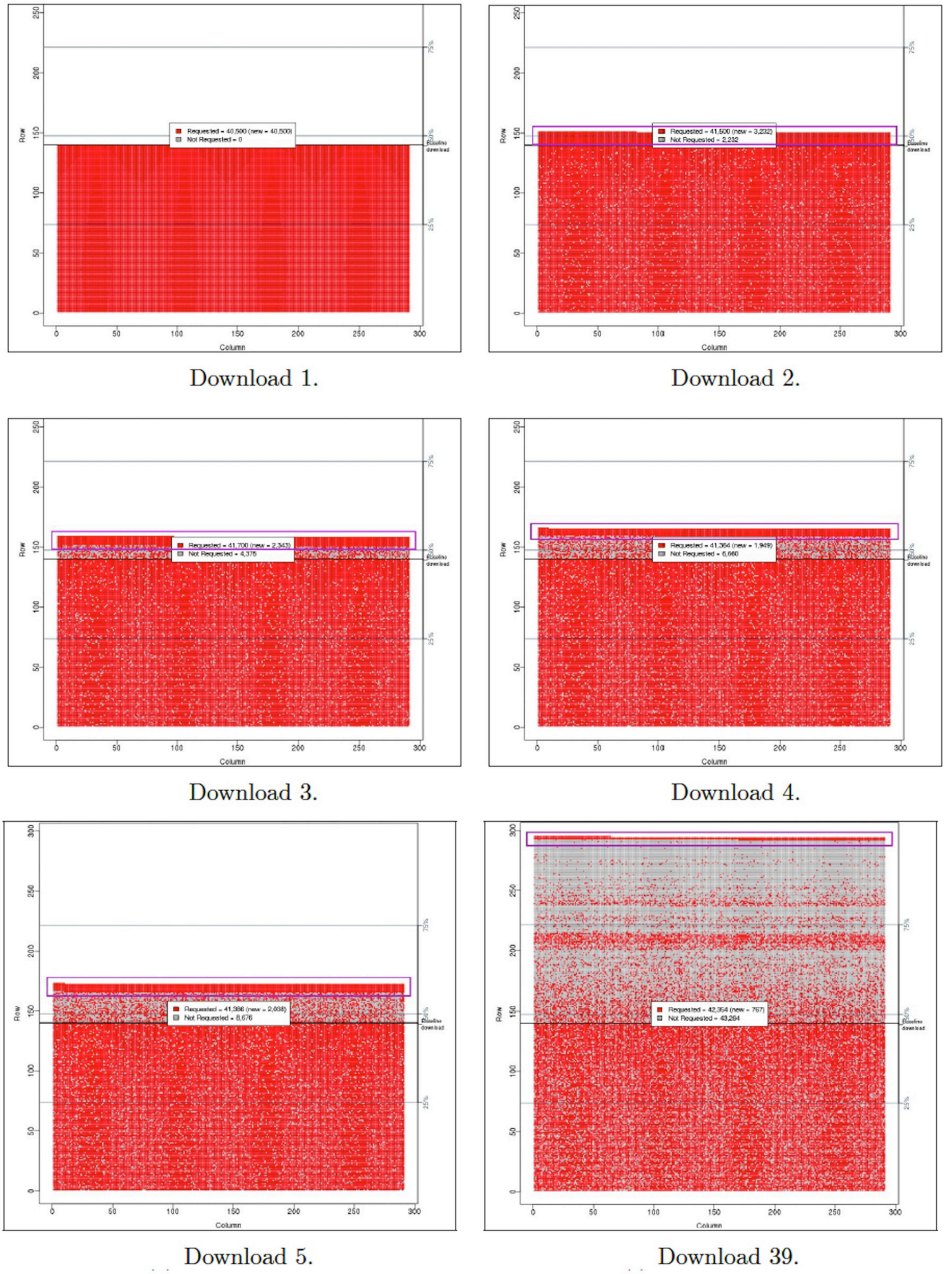
Hash calculations for the resources from 1,566 composite mementos from the Internet Archive in downloads 1–5 and download 39. Each point = hash(HTTP response headers, HTTP entity body, HTTP status code, URI-M). Red = the hash value was observed in this download, Gray = the previously seen hash value was not observed in this download. M. Aturban, A Framework for Verifying the Fixity of Archived Web Resources, PhD dissertation, 2020.

To further investigate the amount of change seen on each download, we constructed an image indicating the unique hash values calculated each time we download the set of mementos from an archive. Fig 25 is an example showing the hash values calculated for each resource over all 1,566 URI-Ms from the Internet Archive for download 1. Each unique hash value is represented by a square in the figure. The squares are stacked such that the square in the bottom left corner represents the hash of the first resource that was downloaded. In download 1, 40,500 unique hashes were calculated. The figure indicates that less than 50% of all the unique hashes that will be calculated over the 39 downloads were seen in download 1. Fig 26 shows snapshots of an animation of this figure over all 39 downloads. The gray squares indicate that a hash that had previously been calculated was not seen in the current download (i.e., a change had occurred).

S1 Fig is an animated GIF version of Fig 26. We also include animated GIFs in S2–S6 Figs for hashes from archive.is, webarchive.loc.gov, webarchive.proni.gov, webcitation.org, and webarchive.org.uk, respectively. Animated GIFs for all archives in this study are available at https://github.com/oduwsdl/mementos-fixity/tree/master/hashing_techniques/.

**Fig 27:**
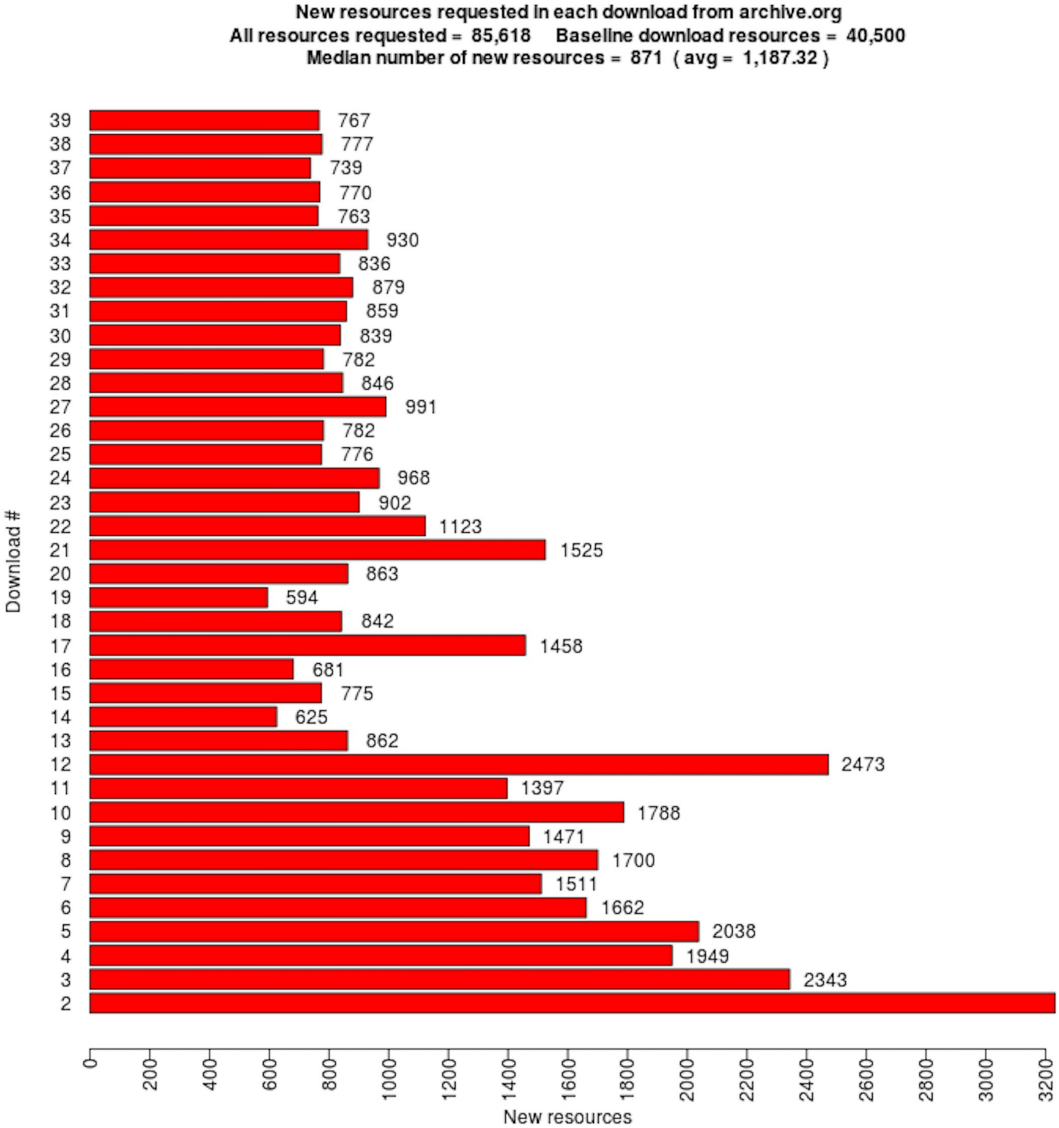
Number of new hash values calculated per download from the Internet Archive. M. Aturban, A Framework for Verifying the Fixity of Archived Web Resources, PhD dissertation, 2020.

Fig 27 shows another way to look at this phenomenon, showing the number of new hash values calculated in each download, starting from download 2 (bottom of the chart).

**Fig 28:**
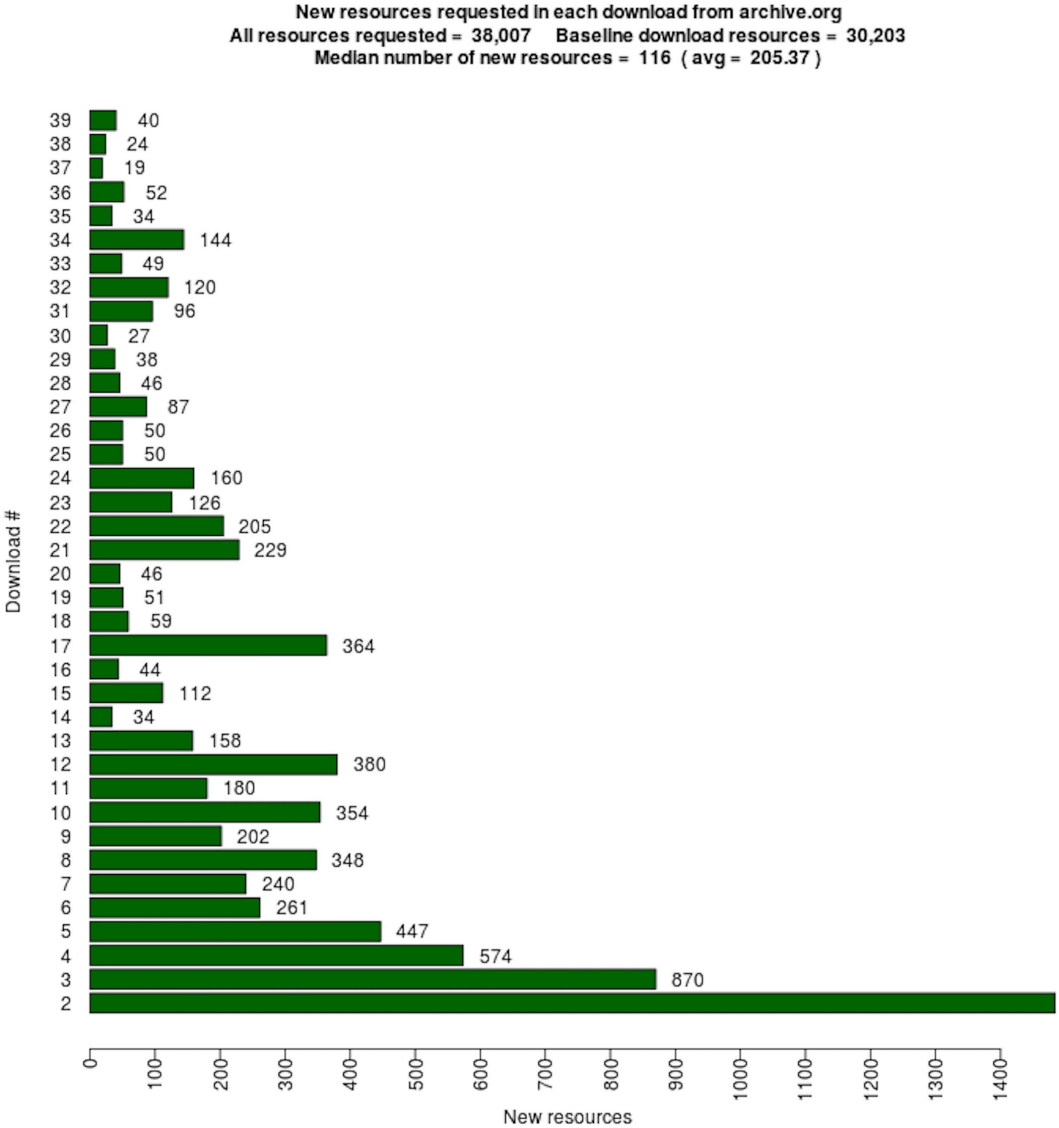
Number of new entities observed per download from the Internet Archive. M. Aturban, A Framework for Verifying the Fixity of Archived Web Resources, PhD dissertation, 2020.

The most lenient policy for detecting changes would be to look only at the resource’s HTTP entity body and use that as the basis for calculating the hash values. Fig 28 shows the number of new unique entities encountered in each download. Even with this lenient policy, there are still a large number of new entities found. Remember that the ideal case would be 0 new entities discovered on subsequent downloads. In S7–S12 Figs, we include animated GIFs showing figures similar to Fig 26 for entity-based hashing for hashes (or, entities) from archive.is, webarchive.loc.gov, webarchive.proni.gov, webcitation.org, and webarchive.org.uk, respectively.

## Discussion

Our 14-month study of the replay of over 16,000 mementos has shown that almost 90% of the mementos produced at least two different hash values, which we presume to be contrary to the expectations of non-expert users of web archives. This is despite our efforts to prevent archive-injected and other live web resources from affecting the computed hash. Our approach to computing hash values for mementos in this work took into account our preliminary study [107] on how to carefully calculate fixity information for composite mementos:

1. Generate fixity information on a composite memento, including the fixity information for each resource embedded in the composite memento
2. Exclude archive-specific content (e.g., archival banners)
3. Use the original HTTP entity bodies and headers, if available
4. If raw mementos are not available, extract original content from rewritten mementos
5. Verify that archives reply with the actual original content in response to requests for raw mementos
6. Avoid mementos served from cache
7. Exclude resources with incorrect HTTP status codes (e.g., server returns HTTP 200 OK for a memento with an archived HTTP 404 status code)
8. Include selected HTTP response headers in hash calculation
9. Exclude any resources from the live Web

Even with these suggested guidelines, there are additional considerations that should be taken into account. First, we have demonstrated that many archived web pages are in flux, meaning that the exact resources loaded by a composite memento at replay time may depend upon the availability of the archive’s holdings, which can be subject to available computation and storage resources, as well as accession and policy changes. Related to this, fixity information computed for mementos that experience transient errors on replay should not be used for comparison. Further, because of the effect of JavaScript and changes in TimeMaps, there may be more than one correct aggregated hash value calculated on the playback of a composite memento over time. For example, in the composite memento shown in Figs 10 and 11, a set of three hash values could cover all possible states. These sets of hashes could be stored in other web archives [89], or in a blockchain [82, 84] or other distributed ledgers. Client-side archiving tools (e.g., ArchiveWeb.page [108]) could be used to create a WACZ file by recording multiple reloads of a web page to ensure inclusion of all of the resources needed by multiple replay attempts of the composite memento in playback tools (e.g., ReplayWeb.page [109]). However, this potential solution would not work for composite mementos that produce different hashes on every replay, such as those indicated by the red, rightmost column in Fig 20.

Unfortunately, a number of challenges prevent the application of conventional hashing techniques, such as blockchain. As we have shown, the archived pages are frequently different on each replay, so it is not always possible to agree on what the inputs for hashing functions should be; there is not even a clear baseline (e.g., first replay) with which we can compare. Large-scale auditing of general web pages will be difficult to automate because of JavaScript embedding different resources, the web archives’ holdings changing (either through patching the archive or regular ingest and deaccession), new releases of replay engines and related infrastructure (web servers, caches, and proxies), as well as transient errors in the network transport. Unfortunately, at scale, the engineering realities of constant churn of changed and different resources on each replay can be impossible to distinguish from malicious manipulation of the archives’ holdings themselves.

Since conventional hashing techniques cannot be used to verify the fixity on the playback of mementos, we should consider new approaches, or *archive-aware hashing*, for generating repeatable fixity information on the playback of archived web pages. This would need to take into consideration the fact that it is likely impossible for an archive replay system both to allow JavaScript execution *and* to have deterministic replay of archived composite mementos. The findings presented in this paper indicate that any future system should consider our suggestions, however they are based largely on the particular mementos that we observed. We do not have an exhaustive exploration of all of the different types of mementos that might be available, so more criteria may be required.

One potential approach for verifying fixity on composite mementos could include client-side replay systems, such as InterPlanetary Wayback (IPWB) [110, 111]. IPWB is a web archive replay system based on InterPlanetary File System (IPFS) [112] that promises replay of the original raw mementos with minimal to zero server-side rewriting. The content-addressable nature of the peer-to-peer file system, IPFS, ensures the fixity of the original content. However, some rewriting is inevitable to prevent live-leakage and for faithful replay of archived web, as discussed earlier. IPWB accomplishes this by using a Service Worker [113] module called Reconstructive [114]. This module resides in the web browser and intercepts all the requests in its scope to make necessary changes in the raw memento response received from the archive server. Effectively, it shifts the responsibility of rewriting from the server to the client. The Reconstructive module also comes with an unobtrusive banner [58], a custom HTML element [115], which is injected in the navigational HTML pages by the Service Worker. Such a system has the potential to integrate client-side fixity verification component in the Service Worker module before any rewriting is performed and corresponding report inclusion in the banner. While this system has the potential to integrate a fixity component, it does not eliminate all of the complexities involved in fixity verification of composite mementos.

Even with the difficulty in computing fixity information for replayed composite mementos, we learned a great deal about the 17 public web archives in this study, not the least of which was tracking the movement of four of the 17 web archives [106]. This suggests that there is utility in periodic monitoring of the playback of the same mementos from multiple archives to globally detect such systemic changes, a sort of web archive observatory service. In addition, there is standards work to be done to create a machine-readable method to describe the provenance of archived web pages as they move from one archive to another.

## Conclusions

This study set out to address the following questions:

- What are the types of changes in the playback of composite mementos that prevent or affect generating repeatable hashes?
- How many times does each identified type of change occur?
- For each composite memento, will excluding certain embedded resources that we expect to change over time help to generate repeatable hash values? Would, for example, excluding resources added by the archive produce more consistent hash values?

Conventional hashing techniques can be applied to server-side files: zip, tar, WARC, or WACZ. However, general purpose web archives like the Internet Archive do not allow for download of WARC files, and likely never will. Most archives only permit user access via a replay engine, such as the Internet Archive’s Wayback Machine or PyWB. To enable third party auditing of a web archive, including potentially uncooperative web archives, we must consider pages as rendered through the replay engine.

We conducted a 14-month study on the replay of 16,627 mementos from 17 public web archives. By replaying the same mementos over time and computing Merkle tree based hash values, we were able to identify seven major types of changes that could cause the same memento to produce different hash values. We found changes in the *Set* of resources comprising a composite memento, the *Status* code of one or more resources, HTTP *Headers* that were not expected to change, the *URI-M* of one or more resources due to redirection, the *Representation* of the HTTP entity body in one or more resources, both the *URI-M* and *Representation* of one or more resources when redirection led to a different entity body, and experienced *Timeout* when one or more resources encountered a connection timeout error.

In our study, we attempted to ensure that resources added or modified by archives to facilitate replay were not included in the calculation of fixity information. Even with these considerations, it was not possible to generate stable fixity information on the replay of composite mementos over time. Almost 90% of our studied mementos produced at least two different hash values over the study period, and over 16% of the mementos produced a distinct hash value on each of the 39 replays.

During our study, we also found that four of the archives moved to new hosting locations and archival services during the time period under observation. Other archives upgraded their replay systems, resulting in the inability to accurately compare the fixity of mementos generated before and after the change.

The lessons learned in this study could be used to produce a more archive-aware hashing function that takes into account changes caused by JavaScript and patterns in computed hash values. We also suggested that a web archive observatory could be developed using such techniques to monitor the state of public web archives in a global scale.

## Supporting information

S1 Fig Hash calculations for 1,566 composite mementos from web.archive.org (Internet Archive) in each download. Each frame shows the hashes computed for all resources on a download. Each point = hash(HTTP response headers, HTTP entity body, HTTP status code, URI-M). Red = the hash value was observed in this download, Gray = the previously seen hash value was not observed in this download.
(GIF)

S2 Fig Hash calculations for 1,396 composite mementos from archive.is in each download. Each frame shows the hashes computed for all resources on a download. Each point = hash(HTTP response headers, HTTP entity body, HTTP status code, URI-M). Red = the hash value was observed in this download, Gray = the previously seen hash value was not observed in this download.
(GIF)

S3 Fig Hash calculations for 1,594 composite mementos from webarchive.loc.gov in each download. Each frame shows the hashes computed for all resources on a download. Each point = hash(HTTP response headers, HTTP entity body, HTTP status code, URI-M). Red = the hash value was observed in this download, Gray = the previously seen hash value was not observed in this download.
(GIF)

S4 Fig Hash calculations for 469 composite mementos from webarchive.proni.gov.uk in each download. Each frame shows the hashes computed for all resources on a download. Each point = hash(HTTP response headers, HTTP entity body, HTTP status code, URI-M). Red = the hash value was observed in this download, Gray = the previously seen hash value was not observed in this download.
(GIF)

S5 Fig Hash calculations for 1,585 composite mementos from webcitation.org in each download. Each frame shows the hashes computed for all resources on a download. Each point = hash(HTTP response headers, HTTP entity body, HTTP status code, URI-M). Red = the hash value was observed in this download, Gray = the previously seen hash value was not observed in this download.
(GIF)

S6 Fig Hash calculations for 349 composite mementos from webarchive.org.uk in each download. Each frame shows the hashes computed for all resources on a download. Each point = hash(HTTP response headers, HTTP entity body, HTTP status code, URI-M). Red = the hash value was observed in this download, Gray = the previously seen hash value was not observed in this download.
(GIF)

S7 Fig Entity-based hash calculations for 1,566 composite mementos from web.archive.org (Internet Archive) in each download. Each frame shows the hashes computed for all resources on a download. Each point = hash(HTTP entity body). Green = the hash value (or, entity) was observed in this download, Gray = the previously seen hash value (or, entity) was not observed in this download.
(GIF)

S8 Fig Entity-based hash calculations for 1,396 composite mementos from archive.is in each download. Each frame shows the hashes computed for all resources on a download. Each point = hash(HTTP entity body). Green = the hash value (or, entity) was observed in this download, Gray = the previously seen hash value (or, entity) was not observed in this download.
(GIF)

S9 Fig Entity-based hash calculations for 1,594 composite mementos from webarchive.loc.gov in each download. Each frame shows the hashes computed for all resources on a download. Each point = hash(HTTP entity body). Green = the hash value (or, entity) was observed in this download, Gray = the previously seen hash value (or, entity) was not observed in this download.
(GIF)

S10 Fig Entity-based hash calculations for 469 composite mementos from webarchive.proni.gov.uk in each download. Each frame shows the hashes computed for all resources on a download. Each point = hash(HTTP entity body). Green = the hash value (or, entity) was observed in this download, Gray = the previously seen hash value (or, entity) was not observed in this download.
(GIF)

S11 Fig Entity-based hash calculations for 1,585 composite mementos from webcitation.org in each download. Each frame shows the hashes computed for all resources on a download. Each point = hash(HTTP entity body). Green = the hash value (or, entity) was observed in this download, Gray = the previously seen hash value (or, entity) was not observed in this download.
(GIF)

S12 Fig Entity-based hash calculations for 349 composite mementos from webarchive.org.uk in each download. Each frame shows the hashes computed for all resources on a download. Each point = hash(HTTP entity body). Green = the hash value (or, entity) was observed in this download, Gray = the previously seen hash value (or, entity) was not observed in this download.
(GIF)
