# Supplementary figures and images for: Hashes are not suitable to verify fixity of the public archived web
Source: PLoS One. 2023 Jun 9;18(6):e0286879. doi: 10.1371/journal.pone.0286879 (PMC10256179; doi:10.1371/journal.pone.0286879)

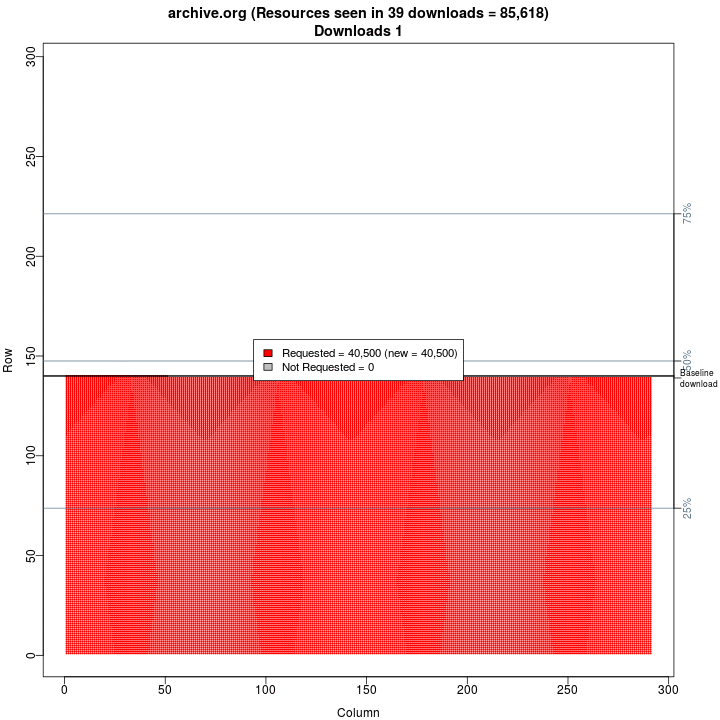

Supplement: S1 Fig — Each frame shows the hashes computed for all resources on a download. Each point = hash(HTTP response headers, HTTP entity body, HTTP status code, URI-M). Red = the hash value was observed in this download, Gray = the previously seen hash value was not observed in this download. (GIF) [file pone.0286879.s001.gif]

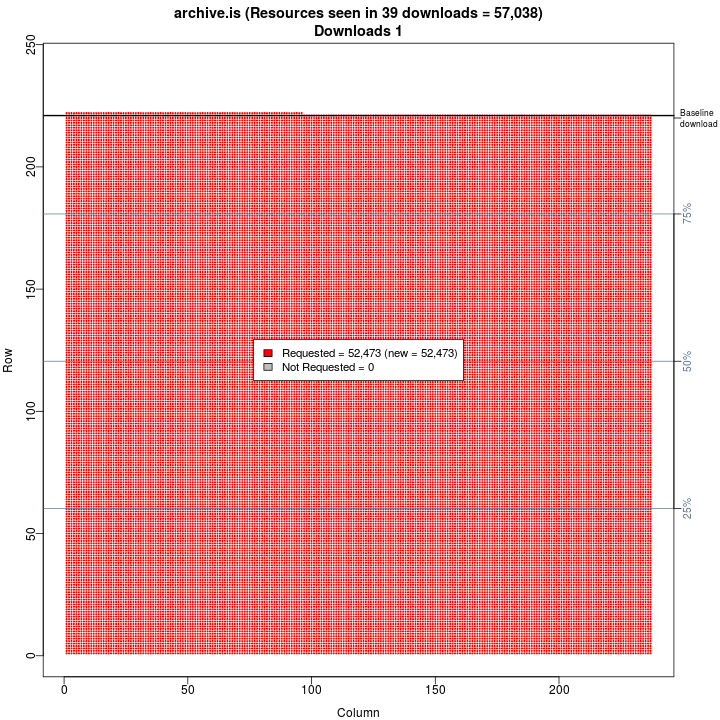

Supplement: S2 Fig — Each frame shows the hashes computed for all resources on a download. Each point = hash(HTTP response headers, HTTP entity body, HTTP status code, URI-M). Red = the hash value was observed in this download, Gray = the previously seen hash value was not observed in this download. (GIF) [file pone.0286879.s002.gif]

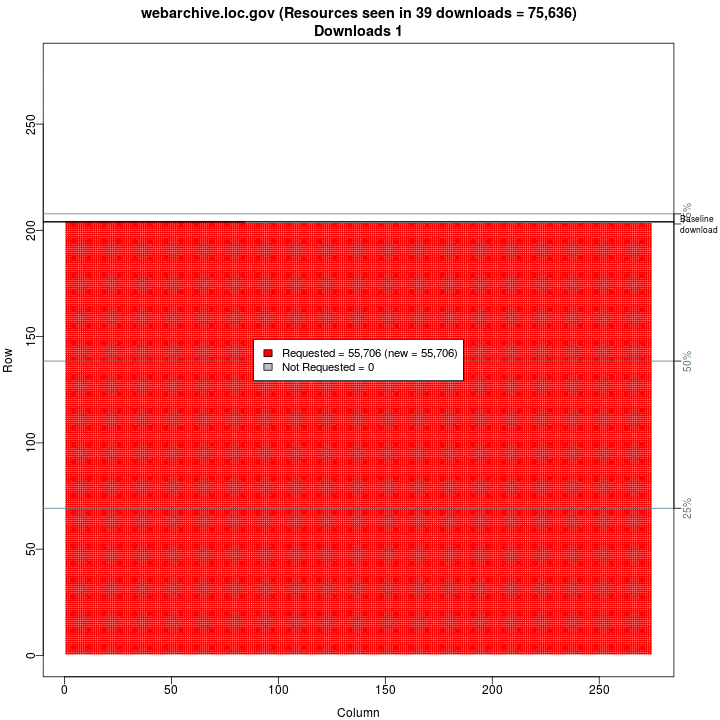

Supplement: S3 Fig — Each frame shows the hashes computed for all resources on a download. Each point = hash(HTTP response headers, HTTP entity body, HTTP status code, URI-M). Red = the hash value was observed in this download, Gray = the previously seen hash value was not observed in this download. (GIF) [file pone.0286879.s003.gif]

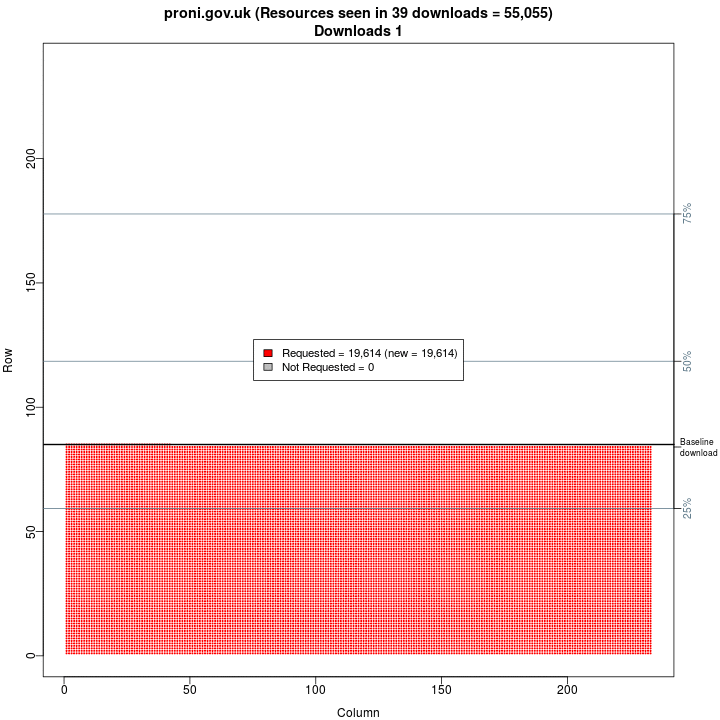

Supplement: S4 Fig — Each frame shows the hashes computed for all resources on a download. Each point = hash(HTTP response headers, HTTP entity body, HTTP status code, URI-M). Red = the hash value was observed in this download, Gray = the previously seen hash value was not observed in this download. (GIF) [file pone.0286879.s004.gif]

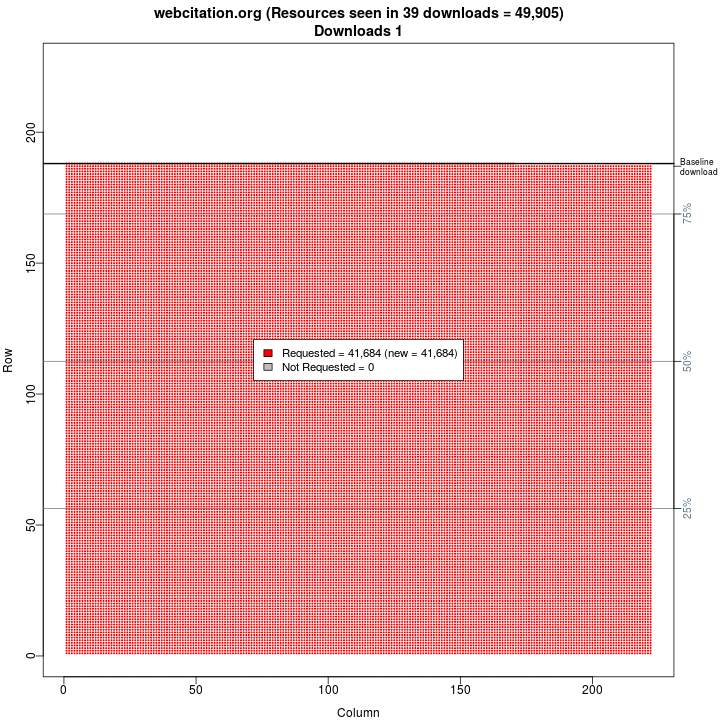

Supplement: S5 Fig — Each frame shows the hashes computed for all resources on a download. Each point = hash(HTTP response headers, HTTP entity body, HTTP status code, URI-M). Red = the hash value was observed in this download, Gray = the previously seen hash value was not observed in this download. (GIF) [file pone.0286879.s005.gif]

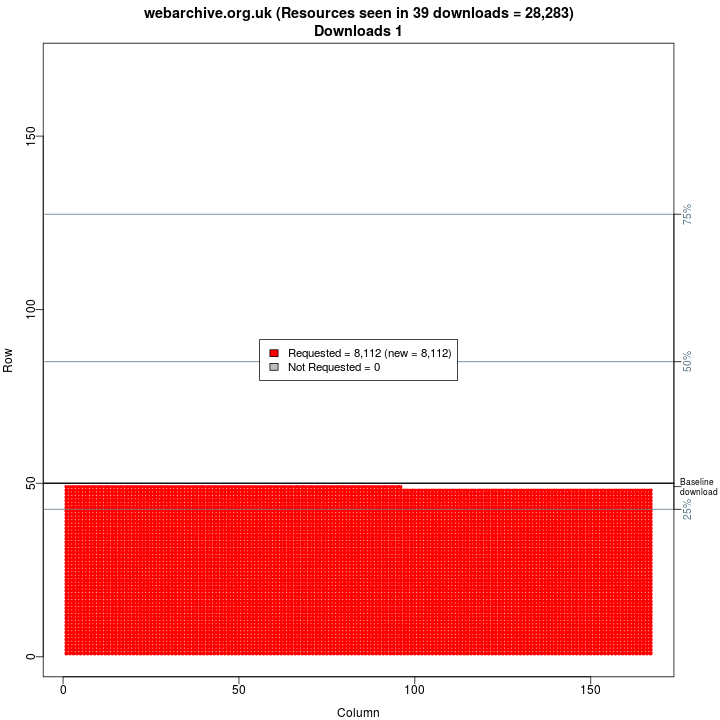

Supplement: S6 Fig — Each frame shows the hashes computed for all resources on a download. Each point = hash(HTTP response headers, HTTP entity body, HTTP status code, URI-M). Red = the hash value was observed in this download, Gray = the previously seen hash value was not observed in this download. (GIF) [file pone.0286879.s006.gif]

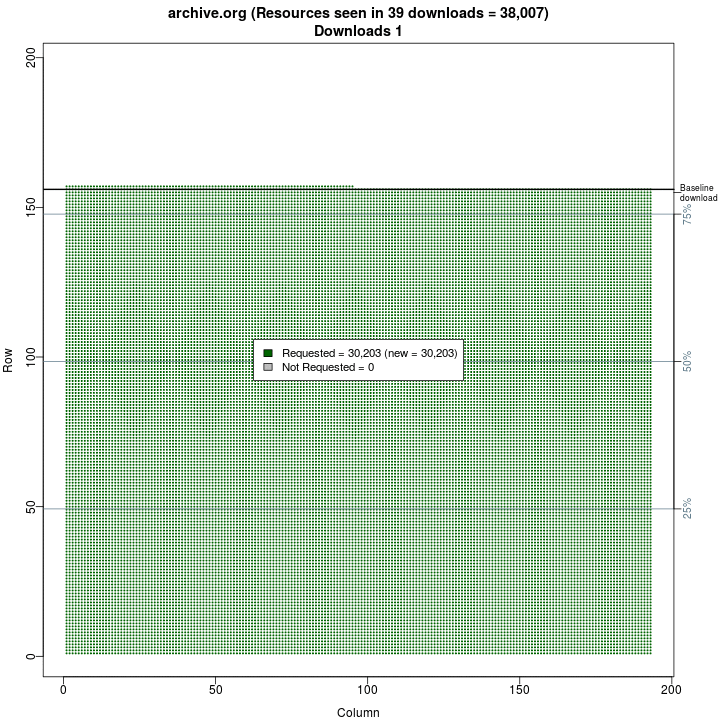

Supplement: S7 Fig — Each frame shows the hashes computed for all resources on a download. Each point = hash(HTTP entity body). Green = the hash value (or, entity) was observed in this download, Gray = the previously seen hash value (or, entity) was not observed in this download. (GIF) [file pone.0286879.s007.gif]

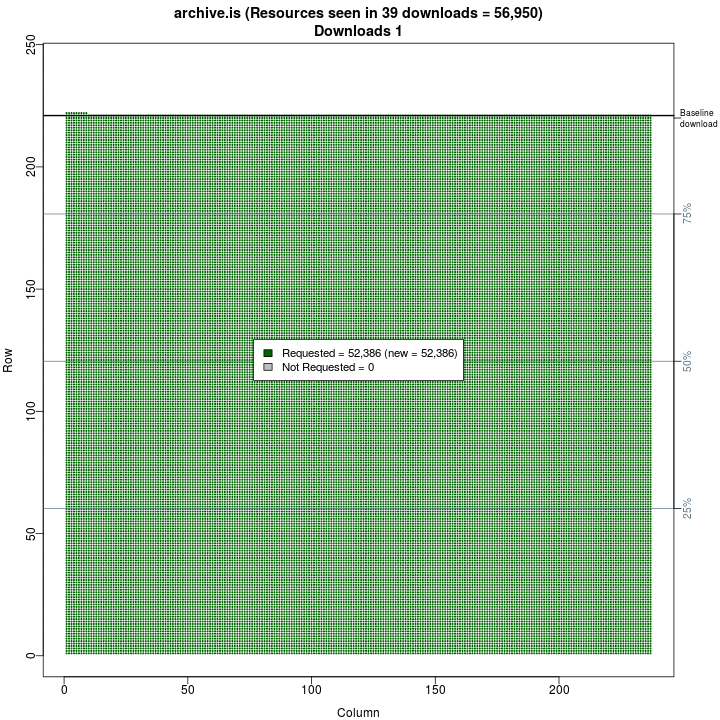

Supplement: S8 Fig — Each frame shows the hashes computed for all resources on a download. Each point = hash(HTTP entity body). Green = the hash value (or, entity) was observed in this download, Gray = the previously seen hash value (or, entity) was not observed in this download. (GIF) [file pone.0286879.s008.gif]

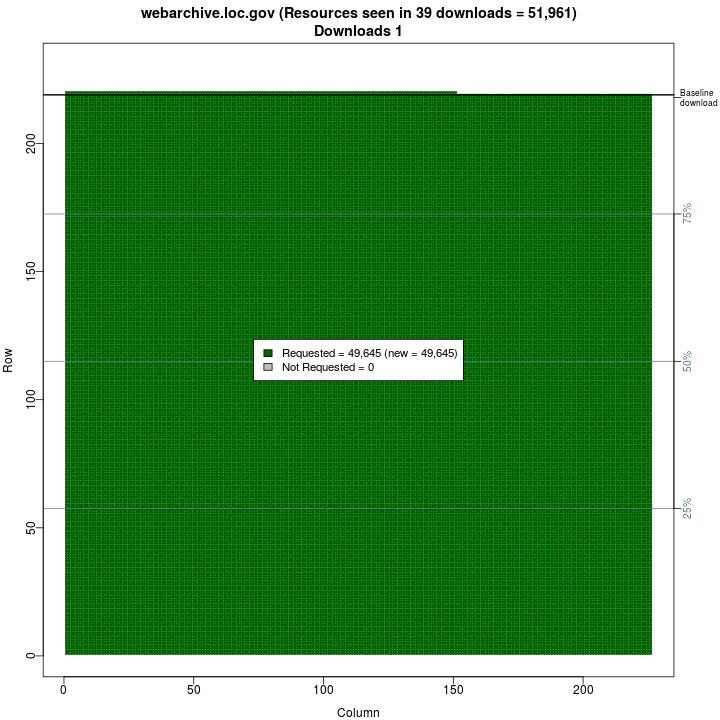

Supplement: S9 Fig — Each frame shows the hashes computed for all resources on a download. Each point = hash(HTTP entity body). Green = the hash value (or, entity) was observed in this download, Gray = the previously seen hash value (or, entity) was not observed in this download. (GIF) [file pone.0286879.s009.gif]

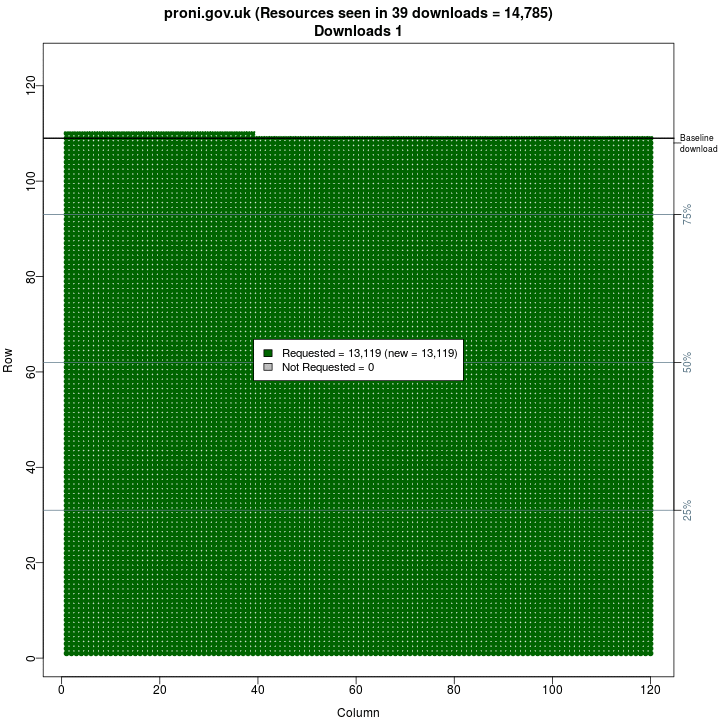

Supplement: S10 Fig — Each frame shows the hashes computed for all resources on a download. Each point = hash(HTTP entity body). Green = the hash value (or, entity) was observed in this download, Gray = the previously seen hash value (or, entity) was not observed in this download. (GIF) [file pone.0286879.s010.gif]

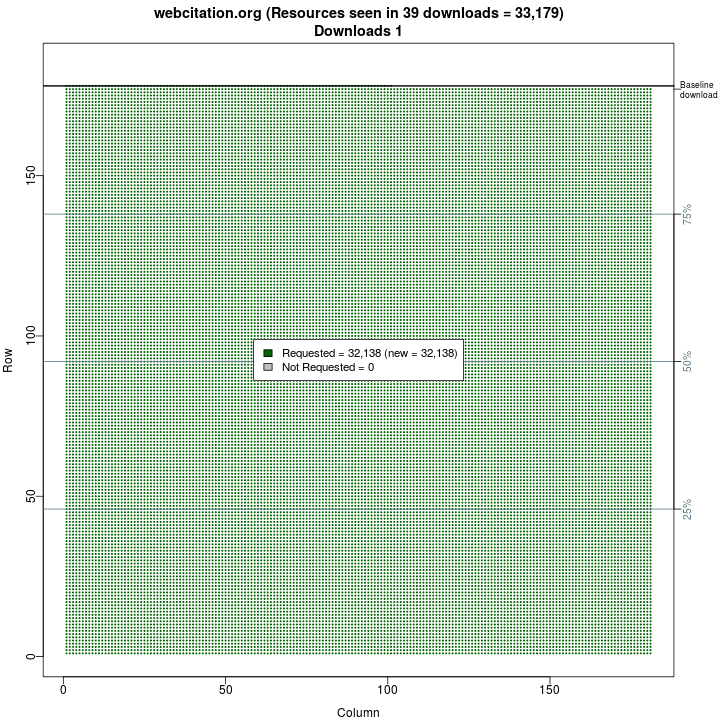

Supplement: S11 Fig — Each frame shows the hashes computed for all resources on a download. Each point = hash(HTTP entity body). Green = the hash value (or, entity) was observed in this download, Gray = the previously seen hash value (or, entity) was not observed in this download. (GIF) [file pone.0286879.s011.gif]

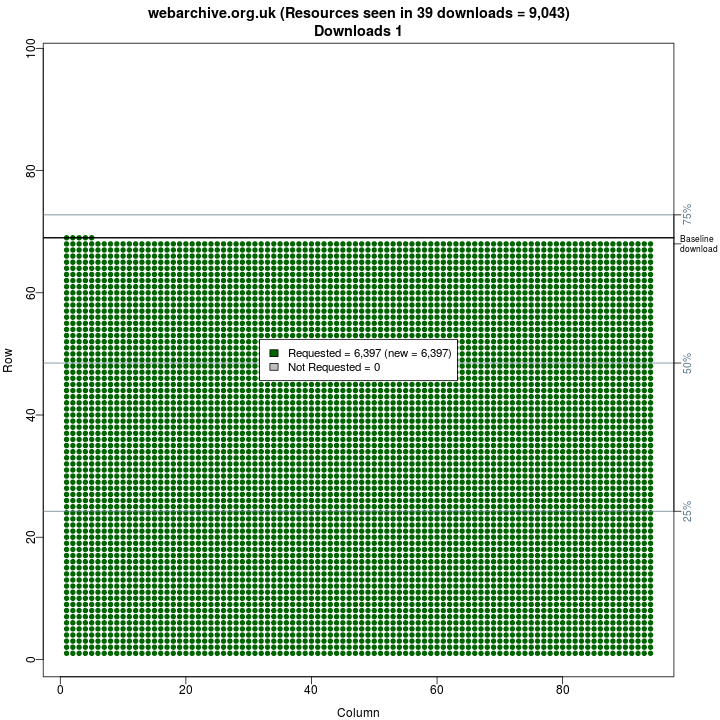

Supplement: S12 Fig — Each frame shows the hashes computed for all resources on a download. Each point = hash(HTTP entity body). Green = the hash value (or, entity) was observed in this download, Gray = the previously seen hash value (or, entity) was not observed in this download. (GIF) [file pone.0286879.s012.gif]
